# Using sagittal otolith morphometrics and shape to discriminate three *Nemipterus* species (Teleostei: Nemipteridae) from Hurghada, Red Sea, Egypt

**DOI:** 10.1038/s41598-026-51812-4

**Published:** 2026-07-01

**Authors:** Imam A. A. Mekkawy, Samia M. El-Mahdy, Usama M. Mahmoud, Ola I. Muhammad

**Affiliations:** 1 Zoology Department, Faculty of Science, Assiut University, Assiut, 71516 Egypt; 2 Fisheries Division, National Institute of Oceanography and Fisheries, NIOF, Cairo, Egypt

**Keywords:** Taxonomy, Size parameters, Shape descriptors, Fourier analysis, SEM, Ecology, Ecology, Evolution, Zoology

## Abstract

Otolith morphology and morphometrics have been widely used to investigate taxonomic distinctions and infer potential relationships among fish species. This study focuses on the sagittal otolith shape and morphological traits of three *Nemipterus* species (*N. zysron*,* N. randalli*, and *N. bipunctatus*), employing size parameters, shape descriptors, Elliptic Fourier Analysis (EFA), and Scanning Electron Microscopy (SEM). Linear relationships were observed between otolith morphometric parameters namely, feret length (OL), feret width (OH), weight (OW), area (OA), and perimeter (OP) and fish biometric traits, including standard length (SL) and body weight (W). Ten shape descriptors, including Circularity (CI), Roundness (RO), and Ellipticity (EL), were evaluated and corrected for size effects. The strongest correlations were noted in OP-OL for *N. zysron* and *N. randalli*, and OA-OL for *N. bipunctatus* Uncorrected descriptors correlated significantly with SL, OL, OH, and OW, whereas corrected descriptors showed mostly non-significant correlations, indicating the effect of size correction. EFA revealed interspecific differences in otolith contour patterns. *N. randalli* and *N. bipunctatus* displayed greater morphological similarity, while *N. zysron* appeared more morphologically distinct. SEM provided detailed structural insights, revealing interspecific differences in the *sulcus acusticus*, rostrum, and margins, with six unique traits distinguishing *N. zysron.* This study demonstrates otolith shape analysis as a cost-effective and reliable method for fish species discrimination. These findings underscore the value of otolith-based approaches as alternatives to genetic marker techniques, enhancing taxonomic research.

## Introduction

The morphology of hard body structures, including otoliths, scales, and bones, has demonstrated utility in distinguishing fish species^1–12^. In teleosts, the inner ear contains three pairs of calcareous otoliths: the sagittae, lapilli, and asterisci^13,14^. These structures exhibit diverse morphological characteristics and contour patterns, with substantial intra- and interspecific variation among marine and freshwater fishes^15,16^. In addition to their taxonomic significance, otoliths provide important insights into the phylogeny and trophic ecology of fish species^17–21^. Otoliths exhibit considerable variation in relative size and shape; however, the sagittae are typically the largest in most teleost fishes and are extensively utilized in taxonomic and biological research^7,8,17,19,22–24^.

The utilization of otolith morphological traits can be classified into three distinct categories. The first category encompasses linear measurements along a single dimension, such as nucleus length, otolith length, hyaline band width, and otolith width^25^. The second category comprises two-dimensional size metrics including area and perimeter, as well as shape indices such as circularity, roundness, ellipticity, rectangularity and aspect ratio^23,26,27^. A third morphological technique examines otolith contours^10,15,28,29^.

Outline analysis provides a detailed understanding of otolith shape variation and variability^30^ and is cost-effective, as it relies solely on images from which shape descriptors can be derived and subsequently analyzed using statistical methods^31^. Currently, several approaches are used to quantify shape, including geometric morphometrics as well as outline-based methods such as Elliptic Fourier Transform and Discrete Wavelet Transform. Among these, the Elliptic Fourier Descriptor (EFD) has been the most widely employed method for otolith shape description in recent decades^10,27,28,32^. This method represents otolith contours using sine and cosine functions to characterize the shape^31^.

Threadfin breams are widely distributed across tropical and subtropical regions of the Indo-West Pacific, constituting a major component of coastal demersal fish assemblages. They play an important role in regional fisheries^33–35^ and have been the subject of increased scientific attention^36,37^. In the last few years, threadfin breams have become one of the most economically important fish resources exploited along the Egyptian waters^38^ especially in the trawl fishery of the Gulf of Suez^39–41^. The catch rate of threadfin bream has risen over the past decade, establishing it as a primary target species, accounting for around 7% of the overall trawl catch in the Suez Gulf^41^. Moreover, the morphological resemblance and similar coloration among *Nemipterus* species pose challenges for species discrimination, underpinning the recent taxonomic recognition of several previously overlooked taxa^42,43^.

Despite the global expansion of otolith research^9,19,28,44–46^, few studies have focused on the family Nemipteridae^17,22^. Moreover, otolith morphology can be influenced by environmental conditions and geographic variation across the distribution range of fish species^47,48^. Accordingly, this study aims to investigate the morphological and shape characteristics of sagittal otoliths distinguishing *Nemipterus zysron*, *N. randalli*, and *N. bipunctatus* from Hurghada, Red Sea, Egypt, employing both traditional morphometric and outline analysis approaches to characterize the otolith features of these species within this region.

## Methods

### Specimen collection

In the present study, a total of 90 specimens of three nemipterid species namely; *Nemipterus zysron*, *Nemipterus randalli* and *Nemipterus bipunctatus* (15 males and 15 females of each species) were collected from Hurghada, Red Sea, Egypt. Species identification was conducted using external morphological characteristics following the diagnostic keys provided in the FAO species identification field guide^35^. Fish standard length (SL) and weight (W) were measured to the nearest 0.1 cm and 1.0 g respectively. Specimens were dissected to extract the left sagittal otoliths, and the sex of each individual was determined.

### Otolith preparation and imaging

The sagittal otolith from the left side was extracted, washed in distilled water, dried, weighed and stored in labeled Eppendorff tubes. All sagittal otoliths extracted were in good condition, showing no visible damage (chips, cracks, bruises, rostrum damage, etc.). For otolith morphometry and shape analysis, left sagittal otoliths were positioned with the outer (lateral) surface facing downward, exposing the mesial surface upward. Two-dimensional digital images were captured using a stereomicroscope equipped with an AxioCam ERc 5s camera (Carl-Zeiss-Promenade 10; 07745 Jena, Germany) at 15 × magnification. Prior to analysis, image calibration was performed by digitizing a stage micrometer under identical imaging conditions, allowing conversion of pixel measurements into metric units (232.46 pixel/mm).

### Otolith shape and morphometric analyses

Traditional morphometry (size parameters & shape descriptors) analysis, Elliptic Fourier analysis (EFA) and scanning electron microscopy were employed for the comparison of otolith shape:

#### Traditional morphometry

**Fig. 1:**
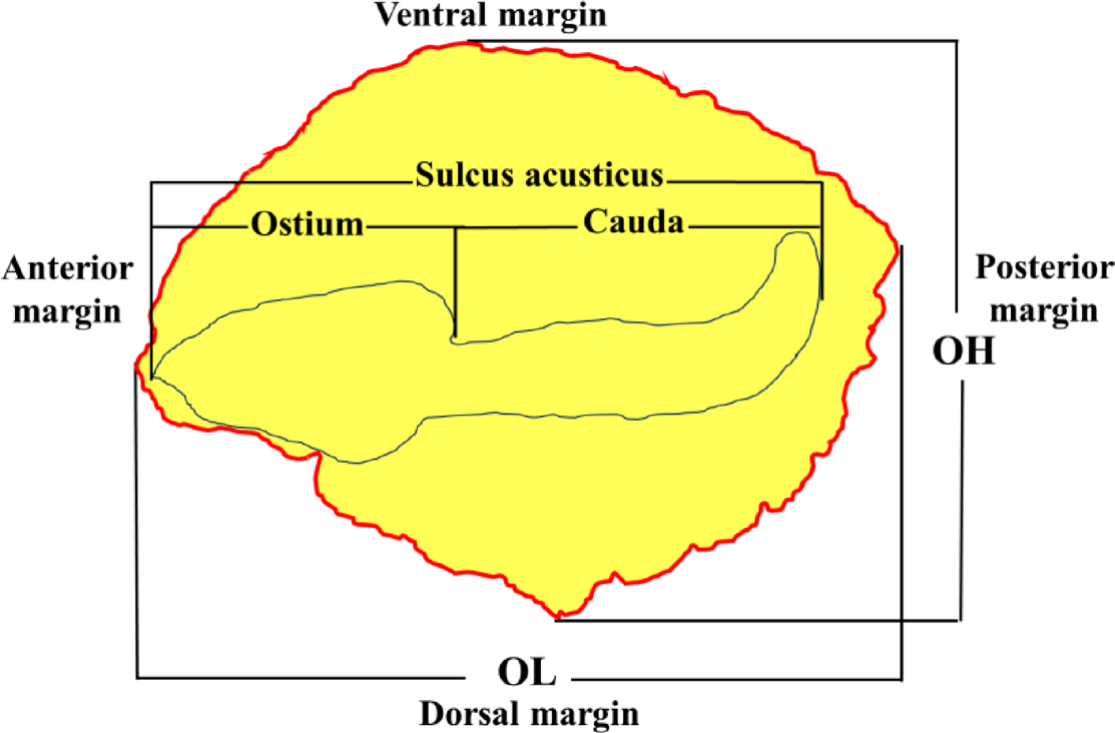
Schematic diagram of the mesial surface of the left sagittal otolith illustrating the definitions of the morphological and morphometric parameters used in the present study: otolith feret length (OL), otolith feret width (OH), otolith area (OA; yellow), and otolith perimeter (OP; red outline).

For the traditional morphometric analysis, otolith images were analyzed and size parameters (Fig. 1) were measured using ImageJ 1.48 V^49^. Those parameters are: Otolith perimeter (OP), Otolith area (OA) and Otolith Feret length (OL) and Otolith Feret width (OH) which represent the length and width of the minimum bounding box that encloses the otolith outline^4^. Ten shape descriptors were quantified: circularity (CI), shape form (SF), eccentricity (EC), ellipticity (EL), roundness (RO), aspect ratio (AR), rectangularity (RE), solidity (SO), compactness (CO) and surface density (SuD), calculated by combining the size parameters in various equations^5,8,34,46,50^ (Table 1). Indicators such as circularity and roundness reflect the resemblance of the otolith contour to a perfect circle, with values ranging from 0 to 1, where 1 represents a perfect circle. Shape form provides insights into the complexity of the otolith contour^50,51^. Ellipticity assesses whether changes in the axes are proportional^22,51^. Eccentricity quantifies the extent to which the otolith outline deviates from circular symmetry by evaluating the spatial distribution of contour points relative to the centroid^52^. Aspect ratio signifies the degree of elongation in the otolith shape, where values greater than 1 indicate an elongated otolith form^22,50^. Rectangularity illustrates the relationship between otolith length and width relative to its area; an RE value of 1 signifies a perfect square, while values below 1 suggest a nearly square shape with minor deviations from symmetry^22,51^. Solidity is a measure of otolith density; a value of 1 indicates a completely solid otolith, while values less than 1 suggest the presence of irregular boundaries or internal cavities^53,54^. Compactness assesses the extent to which otolith material is densely packed within its boundary, often used to measure smoothness or irregularity. Higher compactness values suggest a more regular, smooth outline, while lower values indicate a more irregular or porous structure^8,10,53^. Lastly, Surface density reflects otolith thickness, with higher values indicating greater thickness^51^.

**Table 1:** Size parameters and shape descriptors of the otoliths of the nemipterid species.

Size parameter	Shape descriptors	Equation
Otolith Weight (OW)	Circularity (CI)	$\frac{4 \uppi \mathrm{x} \mathrm{O}\mathrm{A}}{ {\mathrm{O}\mathrm{P}}^{2}}$
	Roundness (RO)	$\frac{4 \mathrm{x} \mathrm{O}\mathrm{A}}{\uppi \mathrm{x} {\mathrm{O}\mathrm{L}}^{2}}$
Otolith area (OA)	Shape form (SF)	$\frac{{\mathrm{O}\mathrm{P}}^{2}}{ \mathrm{O}\mathrm{A}}$
	Ellipticity (EL)	$\frac{\mathrm{O}\mathrm{L} - \mathrm{O}\mathrm{H}}{ \mathrm{O}\mathrm{L} + \mathrm{O}\mathrm{H}}$
Otolith perimeter (OP)	Eccentricity (EC)	$\frac{\mathrm{O}\mathrm{H} }{ \mathrm{O}\mathrm{L}}$
	Aspect ratio (AR)	$\frac{\mathrm{O}\mathrm{L} }{ \mathrm{O}\mathrm{H}}$
Otolith feret length (OL)	Rectangularity (RE)	$\frac{\mathrm{O}\mathrm{A}}{ \mathrm{O}\mathrm{L} \mathrm{x} \mathrm{O}\mathrm{H}}$
	Solidity (SO)	area/convex area
Otolith feret width (OH)	Compactness (CO)	$\frac{4 \uppi \mathrm{x} {\mathrm{O}\mathrm{A}}^{0.5}}{ \mathrm{O}\mathrm{L}}$
	Surface density (SuD)	$\frac{\mathrm{O}\mathrm{W} }{ \mathrm{O}\mathrm{A}}$

#### Elliptic Fourier analysis (EFA)

The shape of sagittal otolith was analyzed using EFA which has been shown to be more effective than those derived from Fast Fourier Transformation (FFT) for shape analysis^55^. This method describes the outline with several components, named harmonics. Each harmonic is characterized by four coefficients, resulting from the projection of each point of the outline on axes (x) and (y). The higher the number of harmonics, the greater the accuracy of the outline description^55,56^. For each numerical image, the software Shape version 1.3^57^ estimated the Fourier.

**Fig. 2:**
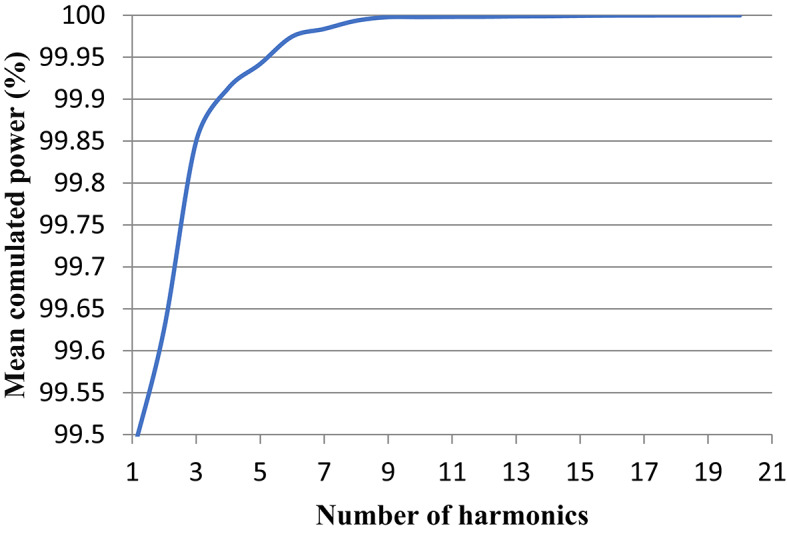
Contribution to the otolith contour description (%) and the number of harmonics used in the present study.

Moreover, the Fourier Power (FP) spectrum was calculated in order to determine the minimum number of harmonics required for the best reconstruction of the otolith outline^58^. The FP of a harmonic is proportional to its amplitude and provides a measurement of the amount of “shape information” described by this harmonic^58^. For the n^th^ harmonic, the Fourier power (FP_n_) is given by the expression:

$${\mathrm{FP}}_{{\mathrm{n}}} = \left( {{\mathrm{A}}_{{\mathrm{n}}}^{2} + {\mathrm{B}}_{{\mathrm{n}}}^{2} + {\mathrm{C}}_{{\mathrm{n}}}^{2} + {\mathrm{D}}_{{\mathrm{n}}}^{2} } \right)/{ 2}$$

where A_n_, B_n_, C_n_ and D_n_ are the Fourier coefficients of the n^th^ harmonic^58^. The cumulated power percentage (PFc) equal the summation of PF_n_ for this purpose. The threshold of 99.99% of the mean cumulated Fourier power was chosen to define the adequate number of harmonics to be considered in the analyses. As the first 20 harmonics totaled 99.99% of the cumulated power (Fig. 2), the Fourier analysis indicated that the otolith shape of the studied species could be summarized by these 20 harmonics, i.e., 80 Fourier coefficients^55^. The software Shape version 1.3 standardizes the size and orientation, giving the first three coefficients with fixed values of A = 1, B = C = 0. Every sagitta was therefore represented by 77 unique coefficients^59^. The principal component values were calculated using the Fourier coefficients in the PrinComp software, which is included in the Shape version 1.3 program package. This software contains four programs (ChainCoder, Chc2Nef, PrinComp, and PrinPrint) that are essential for evaluating the contour shapes based on Elliptic Fourier Descriptors (EFDs) which was first proposed by Kuhl and Giardina^56^.

#### Scanning electron microscopy (SEM)

Scanning Electron Microscopy (SEM) was used to study the morphology and microstructures of the otoliths through the following procedure:

- *Sample preparation* The otoliths were thoroughly cleaned and air-dried prior to analysis. Each specimen was mounted on a holder using adhesive tape to ensure stability during imaging.
- *Coating* the mounted otoliths were coated with a 30-nm layer of gold.
- *Imaging* Electron micrographs were acquired using backscattering mode at an accelerating voltage of 15 kV with a working distance of 28 mm.
- *Equipment details* Imaging was performed on a JEOL JSM-5400LV SEM at the Electron Microscope Unit, Assiut University, Egypt.

Fourteen otolith morphological characters, including overall shape, the lateral and mesial surfaces, as well as the shape of the posterior, dorsal, and ventral margins, rostrum, antirostrum, *sulcus acusticus*, ostio-caudal differentiation, ostium, cauda, crista superior, and crista inferior were examined for each sample. The terminology of otolith morphology follows Smale et al.^60^. The interpretation and description of SEM results were based on previous studies of Jawad et al.^16^, Yedier^61^, Dizaj et al.^19^, Jawad et al.^62^ and Roy et al.^63^.

### Data analysis

The basic statistics for size parameters and shape descriptors (means, standard deviations, and ranges) were computed. The pattern of variation was assessed using a one-way analysis of variance (ANOVA) in SPSS version 22.0^64^ at the 0.05 significance level. Data normality was evaluated using the Shapiro–Wilk test. The homogeneity of variances was tested using Levene’s test (*p* > 0.05, homogeneous variances among samples). Most variables showed homogeneous variances among species (*p* > 0.05); thus, Tukey’s b post-hoc test was applied to examine the interspecific differences in these cases. However, the variables solidity, circularity, shape form and ellipticity did not meet the assumption of equal variances (Levene’s test, *p* ≤ 0.05). For these variables, we applied Tamhane’s T2 post hoc test. Results of this test confirmed those of Tukey’s b post-hoc test due to the low magnitudes of difference between species variances in concern with these variables.

Analysis of covariance (ANCOVAs) of shape descriptors (with SL, OL, OH & OW as covariates) were performed to determine significant relationships between these covariates and interspecific variations reflected by shape descriptor (*p* < 0.05). Such analysis satisfied the corresponding assumptions that combine features of ANOVA and linear regression.

The shape descriptors were corrected to eliminate the influence of body size and allometric effects associated with otolith growth^65^. A power function model, Y_i_ = a X_i_^b^, was applied using a logarithmic transformation to standardize the residuals^66^. Each morphometric index was then transformed into a Z^66^, defined as Z_i_ = Y_i_ (X_0_/X_i_)^b^, where Y_i_ represents the morphometric shape index for each individual, X_i_ is the otolith length of the i-individual, and X_0_ is the mean standard body length (14.8 cm for *N. zysron*, 15.6 cm for *N. bipunctatus*, and 13 cm for *N. randalli*). The value of b for each morphometric index was estimated as the slope of the regression between log Y_i_ and log X_i_^67^.

The relationships between otolith measurements (OL, OH, OW, OA and OP) and SL, W, and OL were analyzed using linear regression models. The strength of each relationship was quantified using the coefficient of determination (R^2^) which ranges from 0 to 1; values closer to 1 indicate a strong relationship, while values near 0 suggest a weak relationship. In addition, correlations between shape descriptors and otolith length, width, weight, and fish standard length were assessed at a significance level of *p* < 0.05. The strength and the direction of these relationships were evaluated using the correlation coefficient (r) which ranges from − 1 to 1, where values close to + 1 indicate a strong positive linear relationship, and values near − 1 indicate a strong negative linear relationship. The sign of r indicates the direction of the relationship.

To estimate the main effects of species and sex and their interaction on the otolith shape descriptors, a two-way ANOVA (Design: Species + Sex + Species*Sex) was conducted using SPSS version 22.0 and the homogeneity of variances was tested using Levene’s test. In cases where heterogeneity of variance was detected, a two-way PERMANOVA was performed using the Euclidean similarity index with 9,999 permutations in PAST version 4.11^68^, to clarify inter- and intraspecific variation in a multivariate context.

To investigate the effects of species, sex, and their interaction on each shape descriptor, two-way ANCOVAs and multivariate analysis of covariance (MANCOVAs) (with OL, OH, and OW as covariates) were performed by SPSS version 22.0 with satisfied assumptions. Discriminant function analysis (DFA) and principal component analysis (PCA) were conducted in PAST version 4.11 for better plotting of interspecific variation. PCA was first applied to the Fourier descriptors using PrinComp software and the effective principal components were retained. These components were subsequently used as input variables in the canonical variate analysis (CVA). To assess the robustness of the results, discriminant analyses were performed using both the full set of Fourier descriptors and the reduced set of effective principal components. The corrected shape indices were analyzed by cluster analysis using Statistica Package Release 8^69^.

Otolith descriptors were further analyzed using stepwise linear discriminant function analysis (LDFA) by SPSS version 22.0 software to determine the shape descriptors responsible for interspecific variation through a jackknifed (leave-one-out) cross-validation procedure.

## Results

In the present study, *Nemipterus zysron* female specimens ranged between 12.2 and 15 cm in standard length (SL) (13.73 ± 1.1) and male specimens ranged between 14.6 and 17.3 cm in SL (15.99 ± 0.9). *Nemipterus bipunctatus* female specimens ranged between 12.9 and 16 cm in SL (14.66 ± 0.9) and male specimens ranged between 14.3 and 18.3 cm in SL (16.57 ± 1). *Nemipterus randalli* female specimens had a range of 9.7–15.5 cm in SL (12.7 ± 1.4) and male specimens ranged between 10.7 and 16.4 cm in SL (13.43 ± 1.4).

### Traditional morphometric analysis

Basic statistics of fish weight, standard length and the otoliths size morphometric parameters (mean ± SD and range) were estimated for the three *Nemipterus* species studied (Table 2). The otolith length (OL), weight (OW), width (OH), area (OA), and perimeter (OP) showed linear relationships with standard length (SL), fish weight (W), and OL in the nemipterid species studied. The strongest linear association was observed between OP and OL in *N. zysron* and *N. randalli*, and between OA and OL in *N. bipunctatus* (Table 3).

**Table 2:** The summary statistics (Mean ± Standard Deviation, Range) of the size parameters of the left sagittal otolith of the three nemipterid species.

Parameters	Species		
	*N. zysron*	*N. bipunctatus*	*N. randalli*
Fish weight (W)	83.23 ± 23.3^A^ (47–126.3)	97.23 ± 24.41^B^ (57–145.3)	64.84 ± 23.36 ^C^ (25–114)
Standard length (SL)	14.86 ± 1.5^A^ (12.2–17.3)	15.63 ± 1.38^A^ (12.9–18.3)	13.06 ± 1.8^B^ (9.7–16.4)
Otolith weight (OW)	0.02 ± 0.004^A^ (0.012–0.029)	0.058 ± 0.01^B^ (0.038–0.076)	0.04 ± 0.01^C^ (0.01–0.07)
Otolith area (OA)	13.42 ± 1.7^A^ (9.44–17.17)	26.7 ± 3.69^B^ (19.38–33.27)	21.37 ± 4.9^C^ (13.62-31.6)
Otolith perimeter (OP)	16.59 ± 1.32^A^ (13.7–18.3)	22.4 ± 1.97^B^ (18.79-26.18)	19.73 ± 2.55^C^ (15.15-24.6)
Otolith Feret length (OL)	5.75 ± 0.42^A^ (4.8–6.38)	7.4 ± 0.52^B^ (6.33-8.5)	6.91 ± 0.89^C^ (5.14-8.44)
Otolith Feret width (OH)	3.45 ± 0.23^A^ (2.83–3.99)	5.07 ± 0.42^B^ (4.29–5.76)	4.4 ± 0.52^C^ (3.51-5.55)

Different letters (A, B, C) indicate statistically significant differences at *p* < 0.05 between species for each parameter. Species that share the same letter are not significantly different from each other.

**Table 3:** Regression relationships between otolith length (OL), weight (OW), width (OH), area (OA) and perimeter (OP) and fish standard length (SL) and weight (W) and otolith length (OL) of the three nemipterid species. R^2^: coefficient of determination.

*N. zysron*		*N. bipunctatus*		*N. randalli*	
Equation	R^2^	Equation	R^2^	Equation	R^2^
OL = 2.1 + 0.24 SL	0.79	OL = 2.5 + 0.32 SL	0.81	OL = 1.11 + 0.44 SL	0.87
OH = 1.6 + 0.12 SL	0.66	OH = 0.97 + 0.26 SL	0.81	OH = 1.15 + 0.24 SL	0.86
OW = − 0.022 + 0.003 SL	0.81	OW = - 0.06 + 0.007 SL	0.86	OW = - 0.03 + 0.005 SL	0.87
OA = 1.28 + 0.98 SL	0.77	OA = - 9.1 + 2.3 SL	0.82	OA = - 10.03 + 2.4 SL	0.89
OP = 4.63 + 0.8 SL	0.83	OP = 3.51 + 1.23 SL	0.87	OP = 3.48 + 1.23 SL	0.87
OL = 4.43 + 0.016 W	0.72	OL = 5.74 + 0.018 W	0.84	OL = 4.66 + 0.034 W	0.83
OH = 2.78 + 0.008 W	0.61	OH = 3.63 + 0.015 W	0.84	OH = 3.13 + 0.02 W	0.81
OW = 0.005 + 0.0002 W	0.76	OW = 0.021 + 0.0004 W	0.84	OW = 0.01 + 0.0004 W	0.90
OA = 8.01 + 0.062 W	0.72	OA = 14.25 + 0.13 W	0.84	OA = 9.17 + 0.18 W	0.89
OP = 12.3 + 0.05 W	0.79	OP = 15.86 + 0.07 W	0.89	OP = 13.33 + 0.09 W	0.82
OH = 0.79 + 0.46 OL	0.74	OH = - 0.53 + 0.75 OL	0.89	OH = 0.61 + 0.55 OL	0.89
OW = − 0.02 + 0.007 OL	0.70	OW = -0.068 + 0.017 OL	0.81	OW = -0.043 + 0.012 OL	0.89
OA = − 8.22 + 3.76 OL	0.89	OA = - 25.12 + 6.94 OL	0.96	OA = -16.10 + 5.42 OL	0.96
OP = − 0.55 + 3 OL	0.92	OP = - 4 + 3.51 OL	0.92	OP = 0.48 + 2.77 OL	0.97

The basic statistics of both the uncorrected and corrected shape descriptors of the otoliths of the studied species are presented in Table 4. The results indicated that the otoliths of *N. zysron* had less circularity (CI) and less roundness (RO) values compared to those of the other two species, while the otoliths of *N. randalli* were less round than those of *N. bipunctatus*. The otoliths of *N. zysron* exhibited higher shape form (SF) values, indicating a more complex contour. In addition, the otoliths of *N. zysron* exhibited a higher ellipticity (EL) values, reflecting more consistent proportions, whereas the otoliths of *N. bipunctatus* displayed lower EL, suggesting greater variability in axis proportions. The otoliths of *N. zysron* also displayed a lower eccentricity (EC) value, indicating a more symmetrical shape, while the otoliths of *N. bipunctatus* had higher EC values, reflecting a more elongated form.

**Table 4:** The summary statistics (Mean ± Standard Deviation, Range) of the uncorrected and corrected shape descriptors of the left sagittal otolith of the three nemipterid species.

Parameters	Species		
	*N. zysron*	*N. bipunctatus*	*N. randalli*
Uncorrected shape descriptors			
Circularity (CI)	0.61 ± 0.03^A^ (0.55–0.69)	0.67 ± 0.04^B^ (0.59–0.75)	0.68 ± 0.03^B^ (0.61–0.75)
Roundness (RO)	0.52 ± 0.03^A^ (0.48–0.6)	0.61 ± 0.02^B^ (0.58–0.65)	0.57 ± 0.02^C^ (0.51–0.66)
Shape Form (SF)	20.6 ± 1.12^A^ (18.19–23)	18.87 ± 1.35^B^ (16.73–21.33)	18.4 ± 0.88^B^ (16.85–20.51)
Ellipticity (EL)	0.25 ± 0.02^A^ (0.2–0.29)	0.19 ± 0.02^B^ (0.16–0.23)	0.22 ± 0.02^C^ (0.17–0.26)
Eccentricity (EC)	0.6 ± 0.02^A^ (0.55–0.66)	0.68 ± 0.02^B^ (0.63–0.72)	0.64 ± 0.02^C^ (0.59–0.7)
Aspect ratio (AR)	1.67 ± 0.07^A^ (1.51–1.81)	1.47 ± 0.05^B^ (1.38–1.58)	1.56 ± 0.07^C^ (1.42–1.71)
Rectangularity (RE)	0.67 ± 0.02^A^ (0.64–0.72)	0.7 ± 0.02^B^ (0.66–0.74)	0.69 ± 0.02^C^ (0.66–0.73)
Solidity (SO)	0.95 ± 0.01^A^ (0.94–0.97)	0.97 ± 0.01^B^ (0.95–0.98)	0.97 ± 0.004^B^ (0.96–0.98)
Compactness (CO)	2.25 ± 0.06^A^ (2.17–2.44)	2.45 ± 0.04^B^ (2.38–2.53)	2.36 ± 0.06^C^ (2.25–2.55)
Surface density (SuD)	0.0015 ± 0.0002^A^ (0.001–0.002)	0.002 ± 0.0002^B^ (0.002–0.003)	0.0017 ± 0.0002^C^ (0.001–0.002)
Corrected shape descriptors			
Circularity (CI)	0.40± 0.02^A^ (0.37–0.45)	0.55 ± 0.038^B^ (0.49–0.62)	0.55 ± 0.02^B^ (0.50–0.63)
Roundness (RO)	0.36 ± 0.01^A^ (0.32–0.40)	0.58 ± 0.019^B^ (0.55–0.62)	0.54 ± 0.02^C^ (0.5–0.6)
Shape Form (SF)	31.0 ± 1.3^A^ (27.96–33.53)	22.84 ± 1.58^B^ (20.23–25.56)	22.71± 0.88^B^ (20.04–25.03)
Ellipticity (EL)	0.38 ± 0.02^A^ (0.34–0.45)	0.17 ± 0.015^B^ (0.14–0.2)	0.20 ± 0.02^C^ (0.15–0.24)
Eccentricity (EC)	0.48 ± 0.01^A^ (0.44–0.52)	0.71± 0.025^B^ (0.66–0.77)	0.67 ± 0.03^C^ (0.61–0.74)
Aspect ratio (AR)	2.07 ± 0.07^A^ (1.94–2.26)	1.42 ± 0.05^B^ (1.29–1.5)	1.50 ± 0.07^C^ (1.35–1.65)
Rectangularity (RE)	0.6 ± 0.01^A^ (0.56–0.62)	0.65 ± 0.013^B^ (0.62–0.68)	0.63 ± 0.01^C^ (0.6–0.66)
Solidity (SO)	0.93 ± 0.007^A^ (0.92–0.95)	0.95 ± 0.005^B^ (0.93–0.96)	0.95 ± 0.005^B^ (0.94–0.96)
Compactness (CO)	1.9 ± 0.04^A^ (1.8–2.0)	2.40 ± 0.04^B^ (2.33–2.48)	2.31 ± 0.05^C^ (2.21–2.47)
Surface Density (SuD)	0.002 ± 0.0002^A^ (0.0014–0.0025)	0.0016 ± 0.0003^B^ (0.0012–0.003)	0.0012 ± 0.0002 ^C^ (0.0006–0.0016)

Different letters (A, B, C) indicate statistically significant differences at *p* < 0.05 between species for each parameter. Species that share the same letter are not significantly different from each other.

Moreover, the otoliths of *N. zysron* had higher aspect ratio (AR) values, suggesting more elongation, while the otoliths of *N. bipunctatus* had lower AR values, indicating less elongation. The rectangularity (RE) of *N. bipunctatus* otoliths was higher, implying a near-square shape, whereas *N. zysron* showed lower RE, suggesting increased irregularity. Similarly, the otoliths of *N. zysron* displayed lower solidity (SO) values compared to the other two species, indicating more irregularity and cavities. The compactness (CO) values were higher for *N. bipunctatus* otoliths, reflecting a more densely packed structure, whereas *N. zysron* otoliths had lower CO, consistent with a more irregular structure. For size-corrected shape descriptors, the otoliths of *N. zysron* exhibited higher surface density (SuD) values than those of the other two species, indicating relatively greater thickness. In contrast, based on uncorrected descriptors, the otoliths of *N. zysron* showed lower SuD values compared with the other species.

The analysis of uncorrected descriptors revealed varying levels of correlation with SL, OL, OH, and OW, with several statistically significant patterns emerging across the studied species.

For SL, CI exhibited significant negative correlations (*p* < 0.05), with *r* = − 0.53 and − 0.49 for *N. zysron* and *N. randalli*, respectively. In contrast, SF demonstrated significant positive correlations (*p* < 0.05), with *r* = 0.5, for *N. zysron* and *N. randalli*. In *N. bipunctatus*, EL, RE, and AR showed significant negative correlations with SL (*p* < 0.05, *r* = − 0. 46, − 0.45 and − 0.46, respectively), whereas EC displayed a significant positive correlation (*p* < 0.05, *r* = 0.46). In *N. randalli*, RE, RO, and CO exhibited significant negative correlations with SL (*p* < 0.05, *r* = − 0.47, - 0.49 and − 0.4, respectively), while, SuD demonstrated significant positive correlations (*p* < 0.05), with *r* = 0.59).

In both *N. zysron* and *N. randalli*, CI, RO, EC, RE and CO displayed significant negative correlations with OL (*p* < 0.05; for *N. zysron*, *r* = − 0.6, − 0.5, - 0.45, - 0.39 and − 0.5; for *N. randalli*, *r* = -0.5,  − 0.63, - 0.44, - 0.51 and - 0.62, respectively), while SF, EL and AR showed a significant positive correlation (*p* < 0.05; for *N. zysron*, *r* = 0.59, 0.45 and 0.44; for *N. randalli* *r* = 0.57, 0.43 and 0.43, respectively). Additionally, SO and RE exhibited significant negative correlations with OL in *N. bipunctatus* (*p* < 0.05, *r* = − 0.4 and − 0.49, respectively), while, SuD exhibited significant positive correlations with OL in N. randalli (*p* < 0.05, *r* = - 0.68).

For OH, the majority of descriptors exhibited non-significant correlations (*p* ≥ 0.05). However, in *N. bipunctatus* and *N. randalli*, CI and RE displayed significant negative correlations (*p* < 0.05, for *N. bipunctatus*, *r* = − 0.37 and − 0.5; for *N. randalli* *r* = − 0.54 and − 0.5, respectively), while SF showed a significant positive correlation (*p* < 0.05, *r* = 0.39 and 0.55, respectively).

In *N. bipunctatus*, SO, EL, and AR exhibited significant negative correlations with OH (*p* < 0.05, *r* =  - 0.42, - 0.6 and - 0.6, respectively), whereas EC demonstrated a significant positive correlation (*p* < 0.05, *r* = 0.6). In *N. randalli*, RO and CO exhibited significant negative correlations with OH (*p* < 0.05, *r* = - 0.38 for both) while, SuD exhibited significant positive correlations with OH (*p* < 0.05, *r* = 0.69).

For OW, the majority of descriptors also showed non-significant correlations (*p* ≥  0.5). Exception includes SuD, which displayed a significant positive correlation across all species (*p* < 0.05, *r* = 0.81, 0.7 and 0.85, for *N. zysron*, *N. bipunctatus* and *N. randalli* respectively). In *N. bipunctatus*, EL and AR exhibited significant negative correlations with OW (*p* < 0.05, *r* = − 0.43 and − 0.42, respectively), while EC showed a significant positive correlation (*p* < 0.05, *r* = 0.43).

In *N. randalli*, CI, RO, RE and CO exhibited significant negative correlations with OW (*p* < 0.05, *r* = - 0.54, - 0.41, - 0.41 and - 0.4, respectively), while SF showed a significant positive correlation (*p* < 0.05, *r* = 0.55).

The analysis of corrected descriptors revealed mostly non-significant correlations with SL, OL, OH, and OW across the studied species, with some notable exceptions:

For SL, all descriptors exhibited non-significant correlations (*p* ≥ 0.05), except for *N. randalli*, where SO, EL, AR and SuD showed significant positive correlations (*p* < 0.05, *r* =  0.49, 0.41, 0.4 and 0.74, respectively), while EC, RO and CO demonstrated a significant negative correlation (*p* < 0.05, *r* = − 0.41, - 0.4 and - 0.39, respectively) and for *N. bipunctatus* where EL, and AR demonstrated a significant negative correlation (*p* < 0.05, *r* = -0.37 and -0.36, respectively).

For OL, most descriptors displayed non-significant correlations (*p* ≥ 0.05). However, in *N. randalli*, SO, EL, AR and SuD exhibited significant positive correlations (*p* < 0.05, *r* =  0.45, 0.55, 0.54 and 0.85, respectively), while EC, RO, and CO demonstrated significant negative correlations (*p* < 0.05, *r* = − 0.55, - 0.54 and - 0.53, respectively). In *N. bipunctatus*, EC exhibited significant positive correlations (*p* < 0.05, *r* = 0.49) while, EL and AR showed significant negative correlations (*p* < 0.05, *r* = -0.5 and -0.49, respectively). In *N. randalli*, SuD exhibited significant positive correlations (*p* < 0.05, *r* = 0.84).

For OH, correlations were predominantly non-significant (*p* ≥ 0.05), except in *N. zysron*, where EC, RO, and CO exhibited significant positive correlations (*p* < 0.05, *r* = 0.51, 0.42 and 0.43, respectively), and EL and AR showed significant negative correlations (*p* < 0.05, *r* = − 0.52 and − 0.52, respectively).

For OW, all descriptors exhibited non-significant correlations (*p* ≥ 0.05) for both *N. bipunctatus* and *N. randalli*, except for SuD, which showed significant positive correlations (*p* < 0.05, *r* =  0.55 and 0.95 respectively). In *N. zysron*, most descriptors also demonstrated non-significant correlations (*p* ≥ 0.05), except for CI, RO, CO, and SuD, which showed significant positive correlations (*p* < 0.05, *r* =  0.46, 0.39, 0.39 and 0.66, respectively), and SF, which exhibited significant negative correlation (*p* < 0.05, *r* = − 0.4). These findings highlight that size correction has mitigated but not fully eliminated the size effect.

ANOVA of corrected descriptors (Design: Species, Sex and interaction) showed that there is an interspecific variation and sexual dimorphism among species studied (*p* < 0.001). Sex has a significant effect on CI and SF, while species*sex has a significant effect on SO, CI, SF, EC, AR and RE. Moreover, Leven’s test of homogeneity was insignificant for some and significant for other descriptors. Therefore, the PERMANOVA test was performed on the otolith descriptors, revealing significant main effects for species, sexes and the interaction factors (*p* < 0.001).

One-way ANCOVAs conducted on the corrected descriptors revealed significant interspecific variation among the species examined (*p* < 0.001). Of the ten otolith descriptors, OL as a covariate significantly influenced EC, RO, AR, EL, CO and SuD. Additionally, OW, OH and SL as covariates had a significant effect only on SuD.

Two-way ANCOVA conducted on the corrected descriptors revealed significant interspecific variation and sexual dimorphism (*p* < 0.001). When OL was used as a covariate, sex had a significant effect on CI, SF, and EC, whereas OL showed no significant effect on SO, RE, CI, and SF. The interaction between species and sex exhibited a non-significant effect on SuD. Using OH as a covariate, both OH and sex had a significant effect on CI and SF, while only OH had a significant effect on SuD. In contrast, the species*sex interaction significantly influenced SO, CI, SF, EC and RE. Similarly, when OW was used as a covariate, both sex and OW significantly affected CI and SF, while only OW had a significant effect on SO and SuD. The species*sex interaction again had a significant effect on SO, CI, SF, EC, AR and RE. Furthermore, the results of the MANCOVA confirmed significant interspecific variation among the studied species. The analysis also demonstrated that sex, the interaction, as well as OL, OH and OW as covariates, had significant effects on the otolith descriptors.

**Fig. 3:**
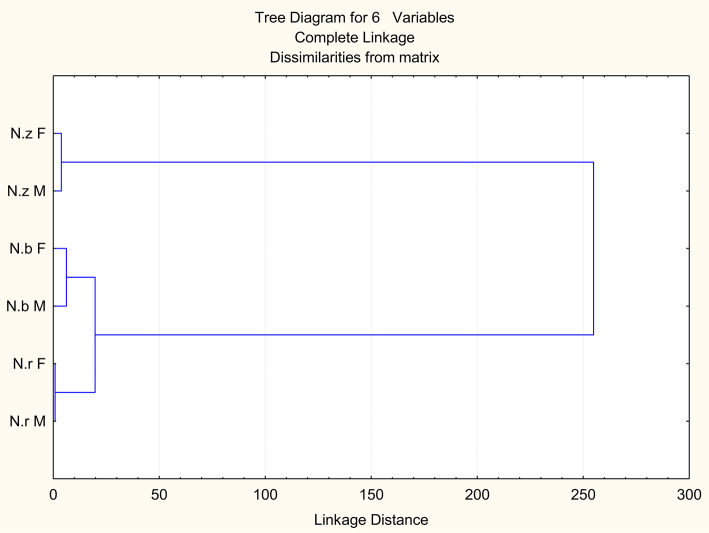
Pattern of sexual dimorphism of three nemipterid species using cluster analysis of otolith shape descriptors based on Squared Mahalanobis Distances (N.z: *Nemipterus zysron*, N.r: *N. randalli*, N.b: *N. bipunctatus*, F: female and M: male).

The pattern of sexual dimorphism in each nemipterid species was evaluated by cluster analysis of the corrected descriptors with clear differences between sex in *N. zysron* versus overlapping in the other two species (Fig. 3).

**Fig. 4:**
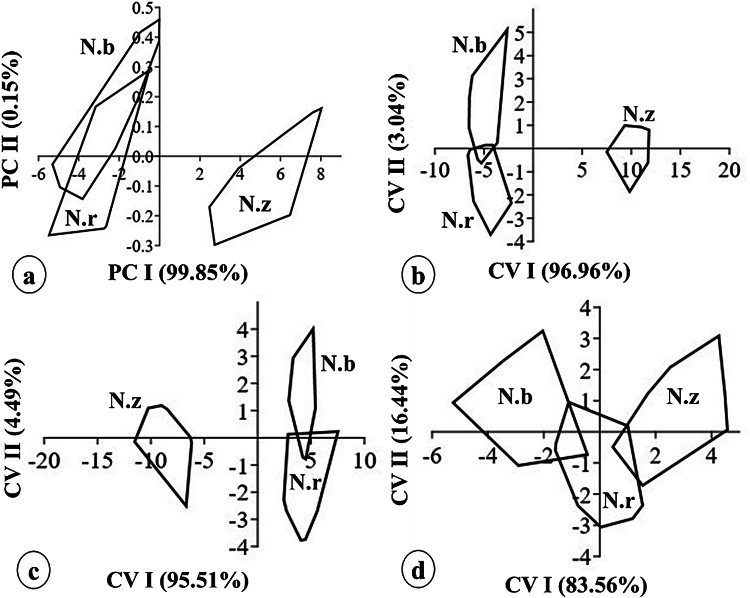
Principal component analysis and discriminant function analysis of otolith shape descriptors for the three nemipterid species (**a** and **b:** size-corrected descriptors, **c:** descriptors determined by Stepwise LDFA and **d**: uncorrected descriptors: N.z: *N. zysron*, N.r: *N. randalli*, N.b: *N. bipunctatus*).

Principal component and discriminant function analysis (LDFA) of the otolith corrected shape descriptors discriminate *N. bipunctatus* and *N. randalli* as one cluster versus *N. zysron* along canonical variate I (CVI); on CVII first two species are slightly overlapped (Fig. 4a and b). Predicted group membership of original cases of *N. zysron*, *N. bipunctatus* and *N. randalli* were 100, 90 & 96.7% respectively with mean of 95.6% of original grouped cases correctly classified. On the other hand, 91.1% of cross validated grouped cases were correctly classified for *N. zysron* (100%), *N. bipunctatus* (86.7%) and *N. randalli* (86.7%) (Table 5).

**Table 5:** Classification success (%) obtained from discriminant function analysis (DFA) using otolith shape descriptors of the studied *Nemipterus* species.

Species	Predicted Group Membership	
	Original cases correctly classified	Cross-validated cases correctly classified
Corrected otolith shape descriptors		
*N. zysron*	100	100
*N. bipunctatus*	90	86.7
*N. randalli*	96.7	86.7
Overall mean	95.6	91.1
Stepwise-selected otolith shape descriptors		
*N. zysron*	100	100
*N. bipunctatus*	90	90
*N. randalli*	93.3	90
Overall mean	94.4	93.3
Un-corrected otolith shape descriptors		
*N. zysron*	86.7	83.3
*N. bipunctatus*	86.7	86.7
*N. randalli*	86.7	76.7
Overall mean	86.7	82.2

Stepwise LDFA referred to SF, RO, EC, CO and SuD to be responsible for the interspecific variations recorded. The pattern of interspecific variations reflected by such analysis was similar to the aforementioned ones (Fig. 4c). Predicted group membership of original cases of *N. zysron*, *N. bipunctatus* and *N. randalli * were 100, 90 & 93.3% respectively with mean of 94.4% of original grouped cases correctly classified. On the other hand, 93.3% of cross validated grouped cases were correctly classified for *N. zysron* (100%), *N. bipunctatus* (90%) and *N. randalli* (90.0%) (Table 5). However, overlapped interspecific variations in nemipterid species were recorded using uncorrected descriptors reflecting the effect of size on both CVI & CVII (Fig. 4d). The classification success (%) obtained from discriminant function analysis (DFA) based on these otolith shape descriptors is presented in Table 5.

### Elliptic Fourier analysis (EFA)

**Fig. 5:**
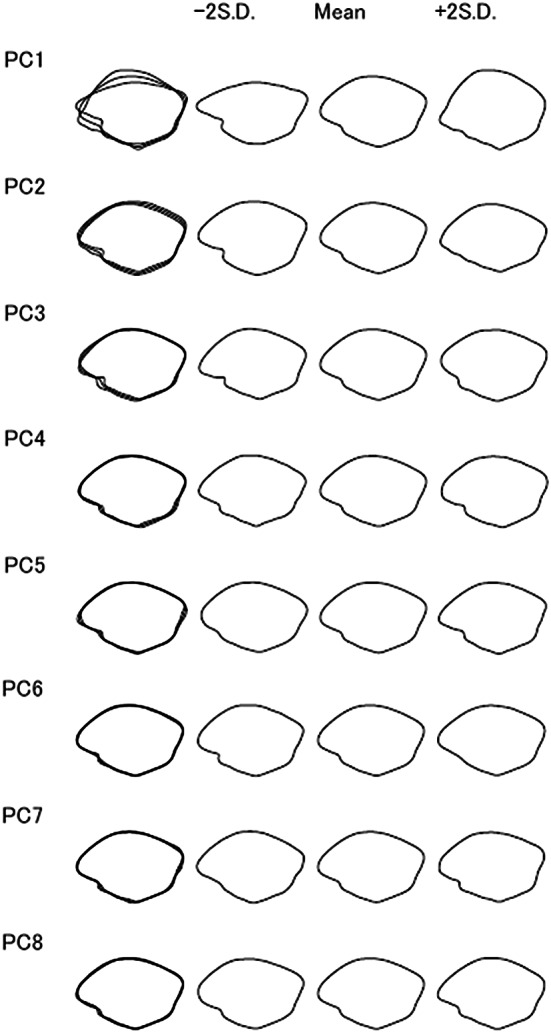
Contours corresponding to different values of the effective principal components (PCs).

The percentages of variance for the effective principal components whose proportion of variance is greater than 1/NAC (where NAC is the number of aligned contour points) of the Elliptic Fourier descriptors are shown in Table 6. In our analysis, out of a total of 77 PCs, 8 were considered effective, based on the threshold criterion (1/77 ≈ 1.30%), collectively explaining 90.73% of the total variance. PC1 is responsible for 53.3% of the overall variation. Figure 5 shows exactly the range and mean of shape patterns reflected by each PC including the degree of elongation of the otolith, the general expression of the rostrum, the antirostrum, the expression of the anterior and posterior margin and the expression of the overall otolith contour.

**Table 6:** Variance of the effective principal components (PC).

Component	Proportion of variance (%)	Cumulative proportion of variance (%)
PC1	53.3	53.3
PC2	16.18	69.48
PC3	8.13	77.61
PC4	4.94	82.55
PC5	2.9	85.45
PC6	2.21	87.66
PC7	1.64	89.31
PC8	1.43	90.73

**Fig. 6:**
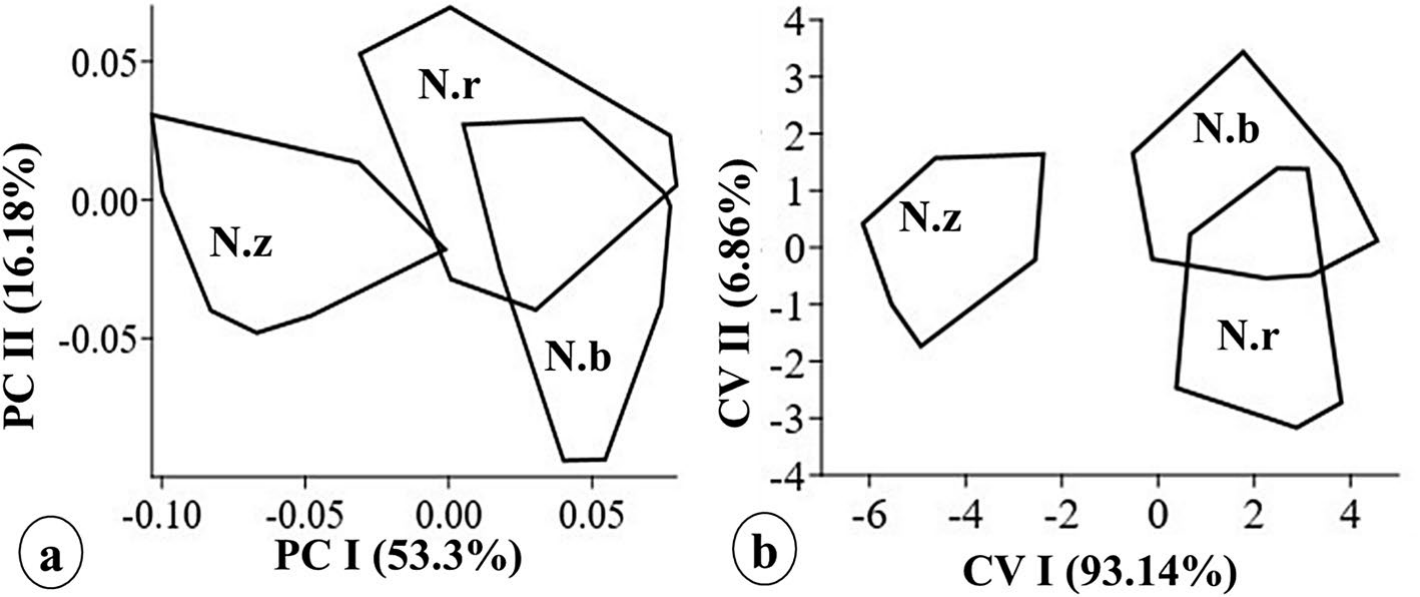
Distribution of samples in the space of principal components (**a**) and the discriminant function analysis based on the Elliptic Fourier descriptors (**b**) of the three nemipterid species. N.z: *N. zysron*, N.r: *N. randalli*, N.b: *N. bipunctatus*.

**Fig. 7:**
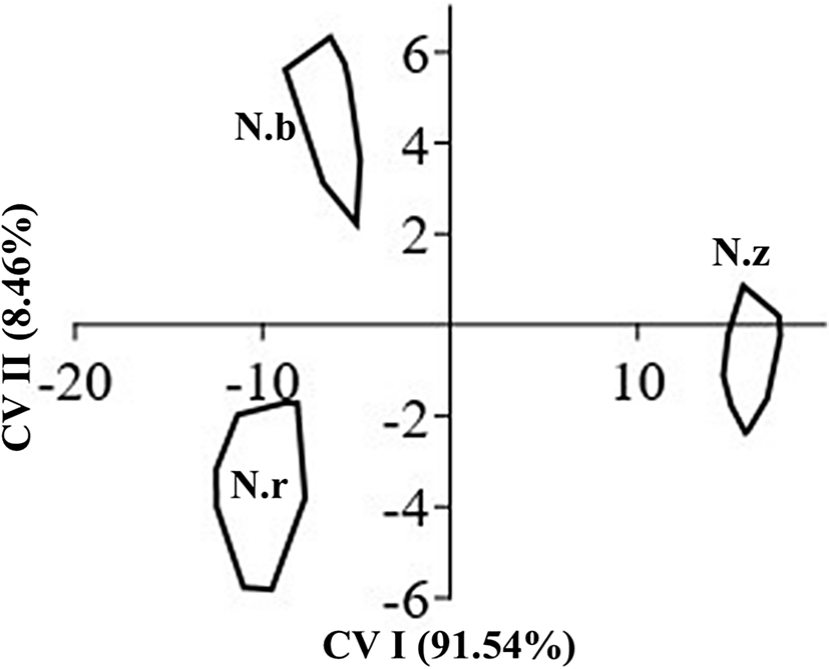
Discriminant function analysis based on the Elliptic Fourier descriptors illustrating the interspecific variation among the three nemipterid species (N.z: *N. zysron*, N.r: *N. randalli*, N.b: *N. bipunctatus*).

The distribution of nemipterid species in concern over the first two PC space (Fig. 6a) revealed that *N. bipunctatus* and *N. randalli* were more similar in the otolith shape accounting for 69.48% of variance (Table 6). Moreover, the PC values obtained were treated by discriminant function analysis (Fig. 6b) indicating a distinct separation of *N. zysron* from the other species along CVI. In contrast, *N. bipunctatus* and *N. randalli* showed partial overlap, although some differentiation between them was observed along CVII. Predicted group membership of original cases of *N. zysron*, *N. bipunctatus* and *N. randalli* were 100, 73.3 & 83.3% respectively with mean of 85.6% of original grouped cases were correctly classified. On the other hand, 82.2% of cross validated grouped cases correctly classified for *N. zysron* (100%), *N. bipunctatus* (70%) and *N. randalli* (76.7%) was estimated (Table 7). Interspecific variations were evident on CVI & CVII using DFA on 77 Elliptic Fourier descriptors (Fig. 7). The classification success (%) obtained from discriminant function analysis (DFA) based on these 77 Elliptic Fourier descriptors is presented in Table 7.

**Table 7:** Classification success (%) obtained from discriminant function analysis (DFA) using otolith Fourier descriptors for the studied species.

Species	Predicted Group Membership	
	Original cases correctly classified	cross-validated cases correctly classified
Effective Fourier descriptors		
*N. zysron*	100	100
*N. bipunctatus*	73.3	70
*N. randalli*	83.3	76.7
Overall mean	85.6	82.2
77 Fourier descriptors		
*N. zysron*	100	86.7
*N. bipunctatus*	100	53.3
*N. randalli*	100	70
Overall mean	100	70

**Fig. 8:**
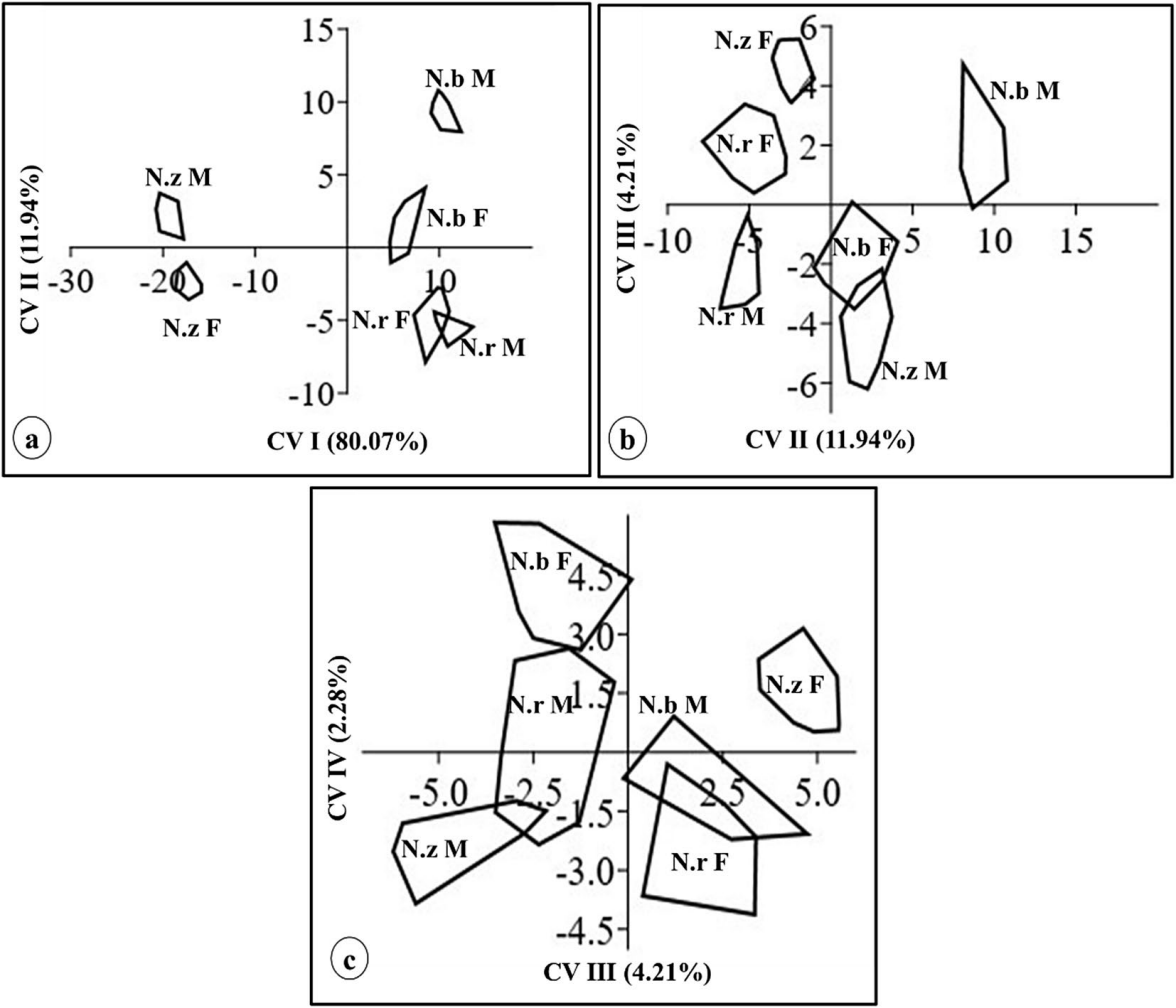
Discriminant function analysis based on the Elliptic Fourier descriptors illustrating the interspecific variations and the sexual dimorphism among the three nemipterid species (N.z: *N. zysron*, N.r: *N. randalli*, N.b: *N. bipunctatus*, F: female, M: male).

**Fig. 9:**
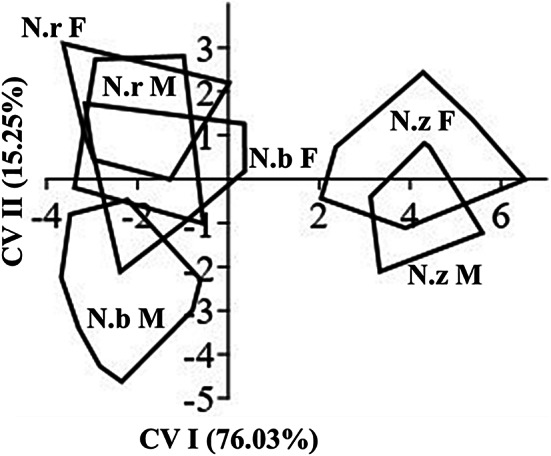
Stepwise discriminant function analysis based on the Elliptic Fourier descriptors illustrating the intra- and inter-specific relationships in nemipterid species (N.z: *N. zysron*, N.r: *N. randalli*, N.b: *N. bipunctatus*, F: female, M: male).

**Fig. 10:**
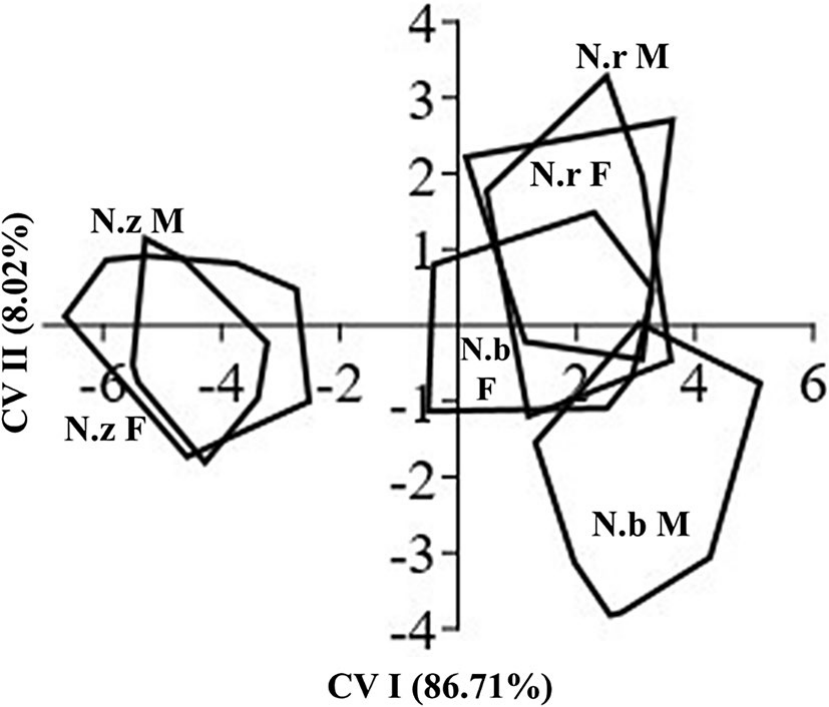
Discriminant function analysis based on the first 8 PC’s of the Elliptic Fourier descriptors of the three nemipterid species (N.z: *N. zysron*, N.r: *N. randalli*, N.b: *N. bipunctatus*, F: female, M: male).

The PERMANOVA test performed on these descriptors revealed significant differences between species, sexes and their interaction (*p* < 0.05). The same pattern was revealed by the PERMANOVA of the first eight principal components (*p* < 0.05). Sexual dimorphism was also evident by application of DFA on 77 Elliptic Fourier descriptors (Fig. 8) since CVI identifies interspecific shape variations whereas CVII and CVIII refer to sexual dimorphism in otolith shape. CVIV represent differences between *N. bipunctatus* female versus the other groups. Predicted group membership of original cases of both sexes of *N. zysron*, *N. randalli* and *N. bipunctatus* indicated a mean classification success of 100% for the original grouped cases. On the other hand, 27.8% of cross validated grouped cases correctly classified for the six sex (female & male) groups of *N. zysron* (40 & 40%), *N. bipunctatus* (26.7 & 33.3%) and *N. randalli* (20 & 6.7%) was estimated Table 8. However, stepwise DFA revealed predicted group membership of original cases with mean of 73.3% versus 63.3% of cross validated grouped cases correctly classified revealing different pattern of relationships on CVI (76.03%) and CVII (15.25%) representing the majority of variations (Table 8; Fig. 9). The first 8 PC’s of these Fourier descriptors revealed interspecific variations between *N. zysron* versus the other species on CVI (86.71%) and only sexual dimorphism of *N. bipunctatus* on CVII representing 8.02% of variance (Fig. 10). DFA of these PC’s indicated predicted sex group membership of original cases with mean of 68.9% versus 54.4% of cross validated grouped cases correctly classified (Table 8).

**Table 8:** Classification success (%) obtained from discriminant function analysis (DFA) using otolith Fourier descriptors for the studied species with respect to sexes.

Species	Predicted Group Membership			
	Original cases correctly classified		Cross-validated cases correctly classified	
	Female	Male	Female	Male
77 Fourier descriptors				
*N. zysron*	100	100	40	40
*N. bipunctatus*	100	100	26.7	33.3
*N. randalli*	100	100	20	6.7
Overall mean	100		27.8	
Stepwise-selected Fourier Descriptors				
*N. zysron*	86.7	86.7	86.7	86.7
*N. bipunctatus*	80	86.7	73.3	73.3
*N. randalli*	40	60	13.3	46.7
Overall mean	73.3		63.3	
Effective Fourier descriptors				
*N. zysron*	80	80	60	66.7
*N. bipunctatus*	73.3	80	73.3	66.7
*N. randalli*	46.7	53.3	20	40
Overall mean	68.9		54.4	

### Scanning electron microscopy

**Fig. 11:**
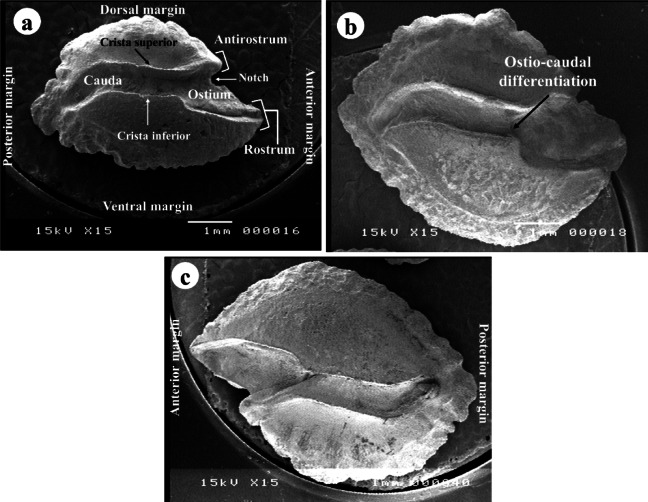
SEM micrographs of left sagittal otoliths from representative specimens of the three nemipterid species. (**a**): *N. zysron* (female, SL = 13.6 cm), (**b**): *N. bipunctatus* (male, SL = 14.2 cm), (**c**): *N. randalli* (female, SL = 13.7 cm).

Interspecific variation in otolith morphology is summarized in Table 9. Two distinct otolith shapes were identified using SEM: an elliptical otolith in *N. zysron* and a pentagonal otolith in *N. randalli* and *N. bipunctatus* (Fig. 11). The lateral surfaces are concave and the mesial surfaces are convex for all the studied species.

**Table 9:** Otolith morphology and microstructure characters of the three nemipterid species.

Character	*N. zysron*	*N. bipunctatus*	*N. randalli*
Overall shape	Elliptic	Pentagonal	Pentagonal
Mesial surface	Convex	Convex	Convex
Lateral surface	Concave	Concave	Concave
Posterior margin	Triangular, lobed with notch	Broadly pointed, irregular	Broadly pointed, lobed
Dorsal margin	Irregular with fine lobes	Irregular	Irregular
Ventral margin	Curved, irregular with fine lobes	Rounded, slightly emarginate	Rounded, slightly emarginate
Rostrum shape	Broadly pointed	Broadly triangular	Triangle
Antirostrum	Pointed	Slightly rounded but shorter	Rounded
*Sulcus acusticus*	Heterosulcoid	Heterosulcoid	Heterosulcoid
Ostio-caudal differentiation	Absent	Present	Present
Ostium	Flared, elongated, deep, floor with ridges, steep walls dorsally and ventrally	Flared, broad, shallow, with irregular floor	Flared, elongated, shallow, floor smooth, steep walls dorsally and ventrally
Cauda	Tubular, deep	Tubular, shallow	Tubular, shallow
Crista superior	Well-developed	Well-developed	Well-developed
Crista inferior	Well-developed	Well-developed	Well-developed

The shape of the posterior margin varied, being triangular and lobed with a notch in *N. zysron*, irregular and broadly pointed in *N. bipunctatus*, and lobed and broadly pointed in *N. randalli*. The dorsal margin ranged from irregular in *N. randalli* and *N. bipunctatus* to irregular with fine lobes in *N. zysron*. The ventral margin varied from curved and irregular with fine lobes in *N. zysron* to rounded and slightly emarginate in *N. randalli* and *N. bipunctatus*.

Three distinct shapes for the antirostrum were identified: pointed in *N. zysron*, slightly rounded and shorter in *N. bipunctatus*, and rounded in *N. randalli*. Two shapes were observed for the rostrum: broadly pointed in *N. zysron* and triangular in *N. randalli* and *N. bipunctatus*, with the rostrum in *N. bipunctatus* being broader.

The *sulcus acusticus* in the studied species is of the heterosulcoid type, characterized by differentiated ostium and cauda on the otolith surface. The ostium is enlarged, while the slender cauda bends downward toward its end. In *N. randalli* and *N. bipunctatus*, the cauda is separated from the ostium by a constriction in the sulcus known as the ostio-caudal differentiation, which is absent in *N. zysron*. In *N. zysron*, the ostium is shorter than the cauda, flared, deep with steep dorsal and ventral walls, and has ridges along the floor. In contrast, the ostium in *N. randalli* and *N. bipunctatus* is flared, shallow, and smooth, with steep walls dorsally and ventrally in *N. randalli*. The cauda is tubular and deep in *N. zysron*, while it is shallower in *N. randalli* and *N. bipunctatus*. The crista superior and crista inferior were well developed in all species studied.

## Discussion

Fish otoliths, particularly the sagittae serve as crucial inner ear structures involved in hearing, balance, and spatial orientation^7^. As a structure that often exhibits species-specific characteristics, the otolith can serve as a useful natural marker, with numerous studies emphasizing the value of its morphological traits for species discrimination and identification^7,8,10,22,29^.

Numerous studies have investigated the relationships between otolith biometry and fish size^17,70–72^. Such relationships can be useful for studies on food and feeding as well as for paleontological studies^70^ where otoliths recovered from the digestive tracts of piscivorous fish can be used to infer the identity and size of consumed species, and total length-otolith measurement studies play a crucial role in understanding prey-predator relationships^24,73^.

While earlier studies primarily focused on assessing fish size in relation to few sagitta measurement, the present study assessed fish size using multiple otolith dimensions. This approach may enhance measurement reliability, as certain otolith features (e.g., rostrum tip or dorsal/ventral edges) may occasionally be damaged, hindering accurate measurement of OL or OW^72^.

The results of this study showed that a linear relationship (R^2^ = 0.79, 0.81 and 0.87 for *N. zysron*, *N. bipunctatus* and *N. randalli*, respectively) existed between sagitta and the fish lengths. Generally, the length of fish exhibits a linear relationship with otolith length. Nevertheless, this relationship is not universally applicable. Otolith length tends to maintain a linear correlation with fish length until the fish reaches its maximum size, after which the otolith primarily increases in thickness^74^.

In addition, linear relationships were observed between body weight and otolith weight in *N. zysron* (R^2^ = 0.76), *N. bipunctatus* (R^2^ = 0.84) and *N. randalli* (R^2^ = 0.89). Hunt^75^ suggested that sagitta otolith weight can serve as a precise and straightforward criterion for estimating fish length and Tuset et al.^5^ further emphasize that otolith weight serves as the most reliable predictor of fish total length, weight, and otolith perimeter.

Previous studies have reported comparable relationships between otolith and fish size measurements. The findings of Innal et al.^76^ indicated that the relationships between OL and fish length, in *N. randalli* from the Antalya Gulf, Turkey, yielded reasonably high coefficients of determination (R^2^); however, these values were lower than those obtained for the same species in the present study. Conversely, the results reported by Uyan et al.^77^ for *N. randalli* from Gökova Bay, Turkey, demonstrated that the relationships between otolith and fish size measurements exhibited R^2^ values similar to those observed in the present study. These similarities further support the consistency of otolith–fish size relationships across different geographic populations. Conversely, observed differences may be attributed to various factors, including discrepancies in measurement techniques due to individual handling and environmental variability among habitats^78^.

Extensive researches have explored the shape descriptors of otoliths across diverse fish species, offering valuable insights into their variability^7,8,10,18,22,51,63,79^. Otolith shape analyses, although being methodologically complex, have demonstrated the potential of otoliths characteristics for species discrimination^4^. Furthermore, the evaluation of otolith shapes has emerged as a cost-effective and efficient approach for distinguishing fish species compared with other techniques, such as genetic marker–based methods^51^.

The relationship between otolith shape descriptors and fish size has been a focal point of research in ichthyology. Previous researches have similarly emphasized the importance of such correlation patterns in various fish species^8,18,71,80^. In this study, the otolith shape descriptors demonstrated different correlations pattern with SL, OL, OH, and OW in the examined species.

The present study reveals distinct interspecific differences in otolith shape descriptors among the three *Nemipterus* species. To date, no prior research has specifically addressed the otolith morphology of *N. zysron* and *N. bipunctatus*, making this study among the first to characterize the species in detail. The otolith of *N. zysron* was relatively elongated, oval-shaped, non-circular, and loosely compact, with sharp features and moderately extended edges. In contrast, *N. bipunctatus* exhibited a relatively circular and compact otolith, with smooth edges and a nearly regular, rounded shape. The otolith of *N. randalli* was semi-circular and slightly more elongated than that of *N. bipunctatus*, but less elongated than *N. zysron*, with edges that are less rounded compared to *N. bipunctatus*.

The development of otoliths occurs under a dual regulation: genetic factors dictate the overall form of the otolith, while environmental conditions, particularly temperature, influence the quantity of material deposited during otolith formation^65,81,82^. This dual regulation highlights the complexity of the biomineralization process, which may also be influenced by factors such as food availability, further contributing to variations in otolith shape descriptors^83^. Reflecting the impact of environmental regulation, SriHari et al.^79^ investigated spatial variations in the stocks of *N. randalli* from five major fishing regions along the Indian coast using otolith shape analyses revealing phenotypic differences among specimens collected from different localities. Similarly, D’Iglio et al.^84^ examined sagittal otolith morphology and morphometry in five Macrouridae species from the Central Mediterranean Sea and reported results that differed from those documented in another geographic region. They attributed these discrepancies to variations in sample size, size class composition, environmental conditions, and species’ life habits among different localities.

However, Sadighzadeh et al.^85^ suggested that genetic differences may represent a major factor influencing otolith shape. In the current study, all *Nemipterus* specimens were collected from the same locality under relatively similar environmental conditions. In addition, the studied species are benthic fishes typically inhabiting sandy or muddy substrates^35^. Under these conditions, environmental variability among samples is expected to be limited, and the observed interspecific differences in otolith morphology may therefore reflect underlying genetic divergence among the studied species.

In this study, LDFA revealed variation in the otolith morphology among the three nemipterid species, with the otoliths of *N. randalli* and *N. bipunctatus* appearing more similar to each other than to those of *N. zysron*. This pattern may indicate a greater degree of morphological divergence in *N. zysron*. A comparable relationship among these species has been reported in molecular phylogenetic study by Ogwang et al.^43^, which suggest that *N. randalli* and *N. bipunctatus* form a closer genetic cluster relative to *N. zysron*.

In the present study, to minimize the influence of size-related variation, shape descriptors were used to standardize otolith measurements. Although juvenile specimens were included (potentially introducing ontogenetic variation that could influence shape) our approach provides a practical basis for species discrimination. Nonetheless, we recommend future studies incorporate detailed maturity or developmental stage data to further control for ontogenetic effects and strengthen species-level morphological comparisons.

The analysis of otolith contour serves as a diagnostic feature for species-level discrimination in fishes^86^. Compared to genotypic methods, EFA offers significant advantages, including reduced time requirements and lower operational costs^87^. This approach effectively captures the full extent of shape variation and subtle individual differences in otolith outlines^7^. Recent studies have demonstrated the efficacy of EFA in characterizing and describing otolith contour shapes^7,10,27,28,51,88^. The greater dissimilarity in otolith shape observed in *N. zysron* compared to *N. bipunctatus* and *N. randalli* suggests that *N. zysron* may be more divergent in terms of otolith morphology. Previous studies have indicated that genetic factors can play an important role in shaping otolith morphology^7,87,89,90^. However, further genetic and phylogenetic analyses are required to confirm the evolutionary relationships among these species.

Otolith shape is known to vary not only among different species but also between sexes^91^. While some studies have reported high discrimination success between sexes using shape descriptors^92,93^, the majority have found these indices to be generally less effective in accurately separating sexes^4,88,94,95^. Our study revealed interspecific variation and sexual dimorphism in otolith shape using both shape descriptors and EFA. In line with our findings, Yazici^88^ reported sexual differentiation in *N. randalli* based on otolith shape descriptors, highlighting the high effectiveness of EFA in distinguishing between males and females.

Former studies investigate numerous characteristics of the sagittal otolith using SEM which help in taxonomic investigations^16,19,61–63,96^. The sagittal otolith of teleost fishes is the most frequently utilized anatomical structure in proportional taxonomic studies employing SEM, owing to its relatively large size and pronounced interspecific variability^62^. However, no studies have specifically addressed the sagittal otoliths of nemipterid species. The findings of this study reveal morphological variations in the otoliths among the three examined species. Among the 14 otolith characteristics analyzed, six traits distinguish *N. zysron* from *N. randalli* and *N. bipunctatus* highlighting their utility for species-level discrimination. Previous studies on both fossil and extant otoliths have shown that sulcus morphology is generally consistent among species within the same genus^97^, which has been interpreted as reflecting a possible genetic basis for this similarity^98^. The findings of the present study indicate that the *sulcus acusticus* morphology of the three examined nemipterid species is largely similar. However, certain morphological differences in the sulcus were observed among the species. Reichenbacher et al.^99^ and Jawad et al.^62^ successfully classified species within the family Aphaniidae (Cyprinodontiformes) and the genus *Lethrinus* (Perciformes), respectively, into groups based on distinct and characteristic sulcus morphologies. Similarly, the observed differences in sulcus morphology within the genus *Nemipterus* in this study suggest the potential for otolith-based classification to define species groups within this genus.

A limitation of the present study is that all specimens were collected from a single locality (Hurghada, Red Sea), and only the left sagittal otolith from each specimen was analyzed. Future studies should incorporate samples from multiple geographic regions to evaluate whether similar patterns of otolith variation are maintained among populations of these species. In addition, analyses including both left and right otoliths would allow assessment of potential bilateral asymmetry in otolith morphology, thereby strengthening the robustness of otolith-based species discrimination.

## Conclusion

This study highlights the significance of otolith analysis for distinguishing fish species within the studied Hurghada, Red Sea population, with particular emphasis on members of the Nemipteridae family. Otolith shape analysis was conducted using an integrated approach that combined traditional morphometric measurements, Elliptic Fourier Analysis (EFA), and scanning electron microscopy (SEM). The results indicate similarities and differences in otolith shapes among the studied species, demonstrating the potential for otolith characteristics to be used as a cost-effective discriminating tool. The results from our analysis also demonstrate inter and intraspecific variations in otolith shape for each species. Furthermore, future research should address the bilateral otolith comparisons, integrating genetic analyses and broader geographic sampling to enhance species discrimination.

## Data Availability

The data supporting this article will be available upon request.

## Abbreviations

EFA: Elliptic Fourier analysis
SEM: Scanning Electron Microscopy
OL: Otolith feret length
OH: Otolith feret width
OW: Otolith weight
OA: Otolith area
OP: Otolith perimeter
SL: Standard length
W: Weight
CI: Circularity
RO: Roundness
SF: Shape form
EL: Ellipticity
EC: Eccentricity
AR: Aspect ratio
RE: Rectangularity
SO: Solidity
CO: Compactness
SuD: Surface density

## References

[1] Jawad, L. A. & Al-Jufaili, S. M. Scale morphology of greater lizardfish *Saurida tumbil* (Bloch, 1795) (Pisces: Synodontidae). *J. Fish Biol.* **70**, 1185–1212. 10.1111/j.1095-8649.2007.01385.x (2007).

[2] Ponton, D. Is geometric morphometrics efficient for comparing otolith shape of different fish species?. *J. Morphol.* **267**, 750–757. 10.1002/jmor.10439 (2006). 16526058 10.1002/jmor.10439

[3] Tuset, V. M., Lombarte, A., González, J. A., Pertusa, J. F. & Lorente, M. Comparative morphology of the sagittal otolith in *Serranus* spp.. *J. Fish Biol.* **63**, 1491–1504. 10.1111/j.1095-8649.2003.00262.x (2003).

[4] Tuset, V. M., Lozano, I. J., Gonzalez, J. A., Pertusa, J. F. & Garcia-Diaz, M. M. Shape indices to identify regional differences in otolith morphology of comber, *Serranus cabrilla* (L., 1758). *J. Appl. Ichthyol.* **19**, 88–93. 10.1046/j.1439-0426.2003.00344.x (2003).

[5] Tuset, V. M., Rosin, P. L. & Lombarte, A. Sagittal otolith shape used in the identification of fishes of the genus *Serranus*. *Fish. Res.* **81**, 316–325. 10.1016/j.fishres.2006.06.020 (2006).

[6] Mekkawy, I. A. A., Mahmoud, U. M., El-Mahdy, S. M. & Essa, F. Morphometrics, geometrics and microstructures of scales of three fish species of genus *Gerres* from the Red Sea, Egypt. *Fish. Sci.*10.1007/s12562-024-01838-2 (2025).

[7] Barani, H. K., Alavi-Yeganeh, M. S., Bakhtiari, A. R. & Ghanbarifardi, M. Otolith shape analysis of five Sillaginidae species from the Persian Gulf and the Oman Sea. *Turk. J. Fish. Aquat. Sci.* **24**, TRJFAS25421. 10.4194/TRJFAS25421 (2024).

[8] Barnuevo, K. D. E. et al. Distinct Stocks of the Redtail Scad *Decapterus kurroides* Bleeker, 1855 (Perciformes: Carangidae) from the Northern Sulu and Southern Sibuyan Seas, Philippines Revealed from Otolith Morphometry and Shape Analysis. *Fishes* **8**, 12. 10.3390/fishes8010012 (2023).

[9] Haimovici, M. et al. Otolith atlas for marine fishes of the southwestern Atlantic occurring along southern Brazil (28° S-34° S). *Mar. Fish Sci.* **37**, 53–207. 10.47193/mafis.3712024010101 (2024).

[10] Morales, C. J. C. et al. Otolith morphometric and shape distinction of three redfin species under the *genus Decapterus* (Teleostei: Carangidae) from Sulu Sea, Philippines. *Fishes* **8**, 95. 10.3390/fishes8020095 (2023).

[11] Park, J. M., Kang, M. G., Kim, J. H., Jawad, L. A. & Majeed, S. Otolith morphology as a tool for stock discrimination of three rockfish species in the East Sea of Korea. *Front. Mar. Sci.* **10**, 1301178. 10.3389/fmars.2023.1301178 (2023).

[12] Mekkawy, I. A. A., Mahmoud, U. M., El-Mahdy, S. M. & Muhammad, O. I. Scale morphometry, geometry and ultrastructure of three *Nemipterus* species from the Egyptian part of the Red Sea. *Sci. Rep.* **15**, 43871. 10.1038/s41598-025-30040-2 (2025). 41398019 10.1038/s41598-025-30040-2 PMC12708734

[13] Bani, A., Poursaeid, S. & Tuset, V. M. Comparative morphology of the sagittal otolith in three species of South Caspian gobies. *J. Fish Biol.* **82**, 1321–1332. 10.1111/jfb.12073 (2013). 23557309 10.1111/jfb.12073

[14] Yedier, S., Bostanci, D. & Türker, D. Morphological and morphometric features of the abnormal and normal saccular otoliths in flatfishes. *Anat. Rec.* **306**, 672–687. 10.1002/ar.25106 (2023). 10.1002/ar.25106 36250249

[15] Afanasyeva, P. K., Orlova, A. M. & Rolsky, A. Y. Otolith shape analysis as a tool for species identification and studying the population structure of different fish species. *Biol. Bull.* **44**, 952–959. 10.1134/S1062359017080027 (2017).

[16] Jawad, L. A. et al. Morphology study of the otoliths of the parrotfish, *Chlorurus sordidus* (Forsska˚l, 1775) and *Hipposcarus harid* (Forsska˚l, 1775) from the Red Sea coast of Egypt (Family: Scaridae). *J. Mar. Biol. Assoc. U K* **98**, 819–828. 10.1017/S0025315416002034 (2017).

[17] Aufy, L. A., Al-Jumaiee, S. A. J., Al-Atbee, I. A. & Al-Mansy, K. A. The relationship between otolith dimensions and fish body size of *Nemipterus japonicus* (Bloch, 1791) in Iraqi marine water. *J. Surv. Fish Sci.* **10**, 5209–5215. 10.17762/sfs.v10i3S.1764 (2023).

[18] Castro-Gutiérrez, J. et al. Exploring morphometric frontiers: A comprehensive study of otolith growth patterns in brown comber *Serranus hepatus* (Linnaeus, 1758). *J. Fish Biol.* **103**, 1374–1381. 10.1111/jfb.15544 (2023). 37641164 10.1111/jfb.15544

[19] Dizaj, L. P., Esmaeili, H. R. & Teimori, A. Comparative otolith morphology of clupeids from the Iranian brackish and marine resources (Teleostei: Clupeiformes). *Acta Zool.* **103**, 29–47. 10.1111/azo.12353 (2022).

[20] Jurado-Ruzafa, A. et al. Phenotypic response of a geographically expanding species, *Scomber colias*: Clues in the fish otolith shape. *Estuar. Coast. Shelf Sci.* **305**, 108880. 10.1016/j.ecss.2024.108880 (2024).

[21] Teimori, A., Iranmanesh, N., Askari Hesni, M. & Motamedi, M. Within-and among-population differentiation of *Aphaniops hormuzensis* from ecologically diverse environments (Cyprinodontiformes; Aphaniidae). *Acta Zool.* **2**, 78 (2020).

[22] Annisa, S. N., Kantun, W. & Kabangnga, A. Otolith shape indices of Japanese Threadfin Bream (*Nemipterus japonicus*, Bloch 1791) from the Makassar Strait, Indonesia. *Asian J. Fish. Aquat. Res.* **26**, 90–96. 10.9734/ajfar/2024/v26i5769 (2024).

[23] Ozpicak, M., Saygin, S. & Polat, N. Otolith shape analysis of bluefish, *Pomatomus saltatrix* (Linnaeus, 1766) in the Black Sea Region (Samsun, Turkey). *Act. Aqua Tr.* **15**, 507–516 (2019).

[24] Yedier, S. et al. Comparison of otolith morphology of invasive big-scale sand smelt (*Atherina boyeri*) from natural and artificial lakes in Turkey. *Iran J. Fish Sci.* **18**, 635–645. 10.22092/ijfs.2018.116980 (2019).

[25] Begg, G. A. & Brown, R. W. Stock identification of haddock *Melanogrammus aeglefinus* on Georges Bank based on otolith shape analysis. *Trans. Am. Fish. Soc.* **129**, 935–945. 10.1577/1548-8659(2000)129<0935:SIOHMA>2.3.CO;2 (2000).

[26] Djamila, I. et al. Use of otolith-shape analysis for stock discrimination of *Boops boops* along the Algerian coast (southwestern Mediterranean Sea). *Afr. J. Mar. Sci.* **39**, 251–258. 10.2989/1814232X.2017.1363817 (2017).

[27] Osman, Y. A. A., Mahé, K., El-Mahdy, S. M., Mohammad, A. S. & Mehanna, S. F. Relationship between fish length and otolith morphological characteristics of *Sargocentron spiniferum* (Forsskål, 1775) from the southern Red Sea. *Oceans* **2**, 624–633. 10.20944/preprints202104.0092.v1 (2021).

[28] Neves, J., Veríssimo, A., Santos, A. M. & Garrido, S. Comparing otolith shape descriptors for population structure inferences in a small pelagic fish, the European sardine *Sardina pilchardus* (Walbaum, 1792). *J. Fish Biol.* **102**, 1219–1236. 10.1111/jfb.15369 (2023). 36880257 10.1111/jfb.15369

[29] Osman, Y. A. A., Pálsson, S. & Makkey, A. F. Otolith shape analysis of *Lethrinus lentjan* (Lacepède, 1802) and *L. microdon* (Valenciennes, 1830) from the Red Sea. *Int. J. Aquat. Biol.* **9**, 159–166. 10.22034/ijab.v9i3.1159 (2021).

[30] Tuset, V. M. et al. Paradox of otolith shape indices: Routine but overestimated use. *Can. J. Fish. Aquat. Sci.* **78**, 681–692 (2021).

[31] Libungan, L. A. & Pálsson, S. ShapeR: An R package to study otolith shape variation among fish populations. *PLoS ONE* **10**, e0121102 (2015). 25803855 10.1371/journal.pone.0121102 PMC4372608

[32] Muniz, A. A. et al. Population structure of the chub mackerel (*Scomber colias*) in the North-east Atlantic inferred from otolith shape and body morphometrics. *Mar. Freshw. Res.* **72**, 341–352 (2021).

[33] Lelli, S., Colloca, F., Carpentieri, P. & Russell, B. C. The threadfin bream *Nemipterus randalli* (Perciformes: Nemipteridae) in the eastern Mediterranean Sea. *J. Fish Biol.* **73**, 740–745. 10.1111/j.1095-8649.2008.01962.x (2008).

[34] Russ, J. C. *Computer-Assisted Microscopy* 1 edn. (Springer, 1990).

[35] Russell, B. C. in *FAO Identification Guide for Fishery Purposes. The Western Central Pacific, 5, Part 3 (Menidae to Pomacentridae).* (ed K.E. Carpenter and V. Niem (eds.)) 3051–3089 (2001).

[36] Aung, T. H. Stock assessment of *Nemipterus japonicus* (Bloch, 1791) in Tha-Bawt-Seik coastal area, Dawei, Myanmar. *J. Fish.*10.17017/j.fish.522 (2024).

[37] Tonie, N., Idris, M. H., Al-Asif, A., Hussin, W. M. R. W. & Kamal, A. H. M. Population characteristics of the Japanese threadfin bream *Nemipterus japonicus* (Bloch, 1791) (Actinopterygii: Nemipteridae) at Bintulu Coast, Sarawak, South China Sea. *Acta Zool. Bulg.* **75**, 273–283 (2023).

[38] El-Haweet, A. E. A. Biological studies of the invasive species *Nemipterus japonicus* (Bloch, 1791) as a Red Sea immigrant into the Mediterranean. *Egypt. J. Aquat. Res.* **39**, 267–274 (2013).

[39] Breikaa, M. I. M. *A study of population dynamics of the threadfin bream Nemipterus japonicus in the Gulf of Suez* M.Sc. thesis, Cairo University, (1992).

[40] Breikaa, M. I. M. *Dynamics and fisheries management of the threadfin bream Nemipterus japonicus (Pisces: Nemipteridae) in the Gulf of Suez* Ph.D thesis, Cairo University, (1996).

[41] Saber, M. A. *Size selection by diamond and square mesh codends for the most commercial demersal fishes in the Gulf of Suez* Ph.D thesis, Ain Shams (2017).

[42] Nakamura, J., Russell, B. C., Moore, G. I. & Motomura, H. *Scolopsis meridiana*, a new species of monocle bream (Perciformes: Nemipteridae) from northern Australia. *Zootaxa* **4500**, 222–234. 10.11646/zootaxa.4500.2.4 (2018). 30486058 10.11646/zootaxa.4500.2.4

[43] Ogwang, J., Bariche, M. & Bos, A. R. Genetic diversity and phylogenetic relationships of threadfin breams (*Nemipterus* spp.) from the Red Sea and eastern Mediterranean Sea. *Genome* **64**, 207–216. 10.1139/gen-2019-0163 (2021). 32678985 10.1139/gen-2019-0163

[44] La Mesa, M. et al. Comparative analysis of otolith morphology in icefishes (Channichthyidae) applying different statistical classification methods. *Fish. Res.* **230**, 105668. 10.1016/j.fishres.2020.105668 (2020).

[45] Lu, Q. et al. Comparative analysis of otolith micro-characteristic in *Schizothorax grahami* and *Spinibarbus sinensis*. *Pak. J. Zool.* **56**, 1185–1192. 10.17582/journal.pjz/20220411130406 (2024).

[46] Qiao, J. et al. Comparative otolith morphology of two morphs of *Schizopygopsis thermalis* Herzenstein 1891 (Pisces, Cyprinidae) in a headwater lake on the Qinghai-Tibet Plateau. *Fishes* **7**, 99 (2022).

[47] Nazir, A. & Khan, M. A. Using otoliths for fish stock discrimination: Status and challenges. *Acta Ichthyol. Pisc.* **51**, 199–218. 10.3897/aiep.51.64166 (2021).

[48] Vaz, A. et al. Otolith shape analysis as a tool for stock identification of two commercially important marine fishes: *Helicolenus dactylopterus* and *Merluccius merluccius*. *Estuar. Coast. Shelf Sci.* **293**, 108471. 10.1016/j.ecss.2023.108471 (2023).

[49] Schneider, C. A., Rasband, W. S. & Eliceiri, K. W. NIH Image to ImageJ: 25 years of image analysis. *Nat. Methods* **9**, 671–675. 10.1038/nmeth.2089 (2012). 22930834 10.1038/nmeth.2089 PMC5554542

[50] Cañás, L., Stransky, C., Schlickeisen, J., Sampedro, M. P. & Fariña, A. C. Use of the otolith shape analysis in stock identification of anglerfish (*Lophius piscatorius*) in the Northeast Atlantic. *ICES J. Mar. Sci.* **69**, 250–256. 10.1093/icesjms/fss006 (2012).

[51] He, T. et al. The use of otolith shape to identify stocks of redlip mullet, *Liza haematocheilus*. *Pak. J. Zool.* **52**, 2265–2273. 10.17582/journal.pjz/20180719080742 (2020).

[52] Duarte-Neto, P., Lessa, R., Stosic, B. & Morize, E. The use of sagittal otoliths in discriminating stocks of common dolphinfish (*Coryphaena hippurus*) off northeastern Brazil using multishape descriptors. *ICES J. Mar. Sci.* **65**, 1144–1152. 10.1093/icesjms/fsn090 (2008).

[53] Wirth, M. A. 97 (University of Guelph, Computing and Information Science, Image Processing Group, 2004).

[54] Jiang, S., Hong, P. & Katayama, S. Impacts of H+ on the otolith morphology of the marbled flounder, *Pseudopleuronectes yokohamae*. *Aquacult. Sci.* **70**, 55–64. 10.11233/aquaculturesci.70.55 (2022).

[55] Lord, C., Morat, F., Lecomte-Finiger, R. & Keith, P. Otolith shape analysis for three *Sicyopterus* (Teleostei: Gobioidei: Sicydiinae) species from New Caledonia and Vanuatu. *Environ. Biol. Fishes* **93**, 209–222. 10.1007/s10641-011-9907-y (2011).

[56] Kuhl, F. P. & Giardina, C. R. Elliptic Fourier features of a closed contour. *Comput. Graph. Image Process.* **18**, 236–258. 10.1016/0146-664X(82)90034-X (1982).

[57] Iwata, H. & Ukai, Y. SHAPE: A computer program package for quantitative evaluation of biological shapes based on elliptic fourier descriptors. *J. Hered.* **93**, 384–385. 10.1093/jhered/93.5.384 (2002). 12547931 10.1093/jhered/93.5.384

[58] Crampton, J. S. Elliptic Fourier shape analysis of fossil bivalves: Some practical considerations. *Lethaia* **28**, 179–186. 10.1111/j.1502-3931.1995.tb01611.x (1995).

[59] Longmore, C. et al. A comparison of otolith microchemistry and otolith shape analysis for the study of spatial variation in a deep-sea teleost, *Coryphaenoides rupestris*. *Environ. Biol. Fishes* **89**, 591–605. 10.1007/s10641-010-9674-1 (2010).

[60] Smale, M. J., Watson, G. &amp; Hecht, T. *Otolith atlas of southern African marine fishes*. Vol. no. 1 (1995:Jun.) (J.L.B. Smith Institute of Ichthyology, 1995).

[61] Yedier, S. Otolith shape analysis and relationships between total length and otolith dimensions of European barracuda, *Sphyraena sphyraena* in the Mediterranean Sea. *Iran. J. Fish. Sci.* **20**, 1080–1096. 10.22092/ijfs.2021.124429 (2021).

[62] Jawad, L. A., Shamsan, E. F., Aguilar, G. & Hoedemakers, K. Scanning electron microscopy and morphological analysis reveal differences in the otolith morphology of three species of the family Lethrinidae (Teleostei: Perciformes) from Yemen. *Anat. Rec.* **306**, 651–664. 10.1002/ar.25115 (2022). 10.1002/ar.25115 36308709

[63] Roy, S., Roy, U. G., Ghorai, N. & Saha, S. K. Developmental variations of sagitta otolith in different body size groups of *Trichogaster fasciata* (Bloch and Schneider, 1801). *Zoomorphology*10.1007/s00435-024-00646-7 (2024).

[64] IBM SPSS Statistics for Windows v. 22.0 (Armonk, NY: IBM Corp, 2019).

[65] Lombarte, A. & Lleonart, J. Otolith size changes related with body growth, habitat depth and temperature. *Environ. Biol. Fishes* **37**, 297–306 (1993).

[66] Lleonart, J., Salat, J. & Torres, G. J. Removing allometric effects of body size in morphological analysis. *J. Theor. Biol.* **205**, 85–93. 10.1006/jtbi.2000.2043 (2000). 10860702 10.1006/jtbi.2000.2043

[67] Jemaa, S. et al. What can otolith shape analysis tell us about population structure of the European sardine, *Sardina pilchardus*, from Atlantic and Mediterranean waters?. *J. Sea Res.* **96**, 11–17. 10.1016/j.seares.2014.11.002 (2015).

[68] Hammer, Ø., Harper, D. A. & Ryan, P. D. PAST: Paleontological statistics software package for education and data analysis. *Palaeontol. Electron.* **4**, 9 (2001).

[69] STATISTICA (data analysis software system) v. 8.0 (StatSoft, Inc. Tulsa, www.statsoft.com, 2007).

[70] Mehanna, S. F., Osman, Y. A. A., Khalil, M. T. & Hassan, A. Relationships between fish and otolith dimensions of *Epinephelus summana* (Forsskål, 1775) and *Cephalopholis argus* (Schneider, 1801) from the Egyptian Red Sea coast. *Egypt. J. Aquat. Biol. Fish.* **23**, 11–21. 10.21608/EJABF.2019.52417 (2019).

[71] Milošević, D., Pešić, A., Ikica, Z., MitroviĆ, T. & Paskaš, N. Biometry of the sagittal otoliths for three demersal fish species from the Eastern Adriatic Sea (Montenegro). *Acta Adriat.* **62**, 171–182. 10.32582/aa.62.2.5 (2021).

[72] Seyfabadi, J., Afshari, M. & Valinassab, T. Otolith morphology and body size relationships of *Nemipterus japonicus* (Bloch, 1791) in the northern Oman Sea. *Indian J. Fish.* **61**, 112–117 (2014).

[73] Aydin, R., Calta, M., Sen, D. & Coban, M. Z. Relationships between fish lengths and otolith length in the population of *Chondrostoma regium* (Heckel, 1843) inhabiting Keban Dam Lake, Pakistan. *J. Biol. Sci.* **7**, 1550–1553. 10.3923/pjbs.2004.1550.1553 (2004).

[74] Radhiah, K., Syazni, K. A., Ismail, N., Khaleel, A. G. & Nasir, S. A. M. Otolith morphology and body size relationships of *Monopterus albus* in Malaysia. *Int. J. Recent Technol. Eng.* **8**, 2662–2667. 10.35940/ijrte.D7277.118419 (2019).

[75] Hunt, J. J. Morphological characteristics of otoliths of selected fish in the Northwest Atlantic. *J. Northwest Atl. Fish. Sci.* **13**, 63–75. 10.2960/j.v13.a5 (1992).

[76] Innal, D. et al. Age and growth of *Nemipterus randalli* from Antalya Gulf-Turkey. *Int. J. Fish. Aquat. Stud.* **2**, 299–303 (2015).

[77] Uyan, U., Jawad, L. A., Filiz, H., Tarkan, A. S. & Çelik, M. Fish length and otolith size of in *Nemipterus randalli* Russell, 1986 (Actinopterygii: Perciformes: Nemipteridae) collected from Gökova Bay, Turkey. *Thalassia Salent.* **41**, 137–146. 10.1285/i15910725v41p137 (2019).

[78] Kasapoglu, N. & Duzgunes, E. The relationship between somatic growth and otolith dimensions of Mediterranean horse mackerel (*Trachurus mediterraneus*) from the Black Sea. *J. Appl. Ichthyol.* **29**, 230–233. 10.1111/jai.12019 (2013).

[79] SriHari, M., Bhushan, S., Nayak, B. B., Pavan-Kumar, A. & Abidi, Z. J. Spatial variations in the stocks of Randall’s threadfin bream, *Nemipterus randalli* Russell 1986 along the Indian Coast inferred using body and otolith shape analysis. *Thalassas Int. J. Mar. Sci.* **37**, 883–890. 10.1007/s41208-021-00309-0 (2021).

[80] De La Cruz-Agüero, J., García-Rodríguez, F. J., De La Cruz-Agüero, G. & Díaz-Murillo, B. P. Identification of gerreid species (Actinopterygii: Perciformes: Gerreidae) from the Pacific coast of Mexico based on sagittal otolith morphology analysis. *Acta Ichthyol Piscatoria*10.3750/aip2011.42.4.03 (2012).

[81] Assis, I. O. et al. Ecomorphological patterns in otoliths of tropical fishes: Assessing trophic groups and depth strata preference by shape. *Environ. Biol. Fishes* **103**, 349–361. 10.1007/s10641-020-00961-0 (2020).

[82] Volpedo, A. & Echeverria, D. D. Ecomorphological patterns of the sagitta in fish on the continental shelf off Argentine. *Fish. Res.* **60**, 551–560. 10.1016/S0165-7836(02)00170-4 (2003).

[83] Manginsela, F. B., Mamuaya, G. E., Lumingas, L. J. & Rompas, R. M. Otolith size and shape index of mackerel scad *Decapterus macarellus* (Cuvier, 1833) from Manado Bay and Kema Bay, North Sulawesi, Indonesia. *AACL Bioflux* **13**, 1723–1734 (2020).

[84] D’Iglio, C. et al. Eco-morphology of sagittal otoliths in five Macrouridae species from Central Mediterranean Sea. *BMC Ecol. Evol.* **25**, 56. 10.1186/s12862-025-02395-7 (2025). 40437356 10.1186/s12862-025-02395-7 PMC12117684

[85] Sadighzadeh, Z. et al. Use of otolith shape for stock identification of John’s snapper, *Lutjanus johnii* (Pisces: Lutjanidae), from the Persian Gulf and the Oman Sea. *Fish. Res.* **155**, 59–63. 10.1016/j.fishres.2014.02.024 (2014).

[86] Salimi, N., Loh, K. H., Kaur, D. S. & Chong, V. C. Fully-automated identification of fish species based on otolith contour: Using short-time Fourier transform and discriminant analysis (STFT-DA). *PeerJ* **4**, 1664. 10.7717/peerj.1664 (2016). 10.7717/peerj.1664 PMC4768690 26925315

[87] Treinen-Crespo, C., Villegas-Hernández, H., Guillén-Hernández, S., Ruiz-Zárate, M. A. & González-Salas, C. Otolith shape analysis as a tool for population discrimination of the white grunt (*Haemulon plumieri*) stock in the northern coast of the Yucatan Peninsula, Mexico. *Rev. Mar. Cost* **4**, 157–168 (2012).

[88] Yazici, R. Sex-linked variations in the sagittal otolith biometry of *Nemipterus randalli* (Russell, 1986) from the eastern Mediterranean Sea. *J. Fish Biol.* **102**, 241–247. 10.1111/jfb.15256 (2023). 36271820 10.1111/jfb.15256

[89] Burke, N., Brophy, D. & King, P. A. Otolith shape analysis: Its application for discriminating between stocks of Irish Sea and Celtic Sea herring (*Clupea harengus*) in the Irish Sea. *ICES J. Mar. Sci.* **65**, 1670–1675. 10.1093/icesjms/fsn177 (2008).

[90] Vignon, M. & Morat, F. Environmental and genetic determinant of otolith shape revealed by a non-indigenous tropical fish. *Mar. Ecol. Prog. Ser.* **411**, 231–241. 10.3354/meps08651 (2010).

[91] Campana, S. E. & Casselman, J. M. Stock discrimination using otolith shape analysis. *Can. J. Fish. Aquat. Sci.* **50**, 1062–1083. 10.1139/f93-123 (1993).

[92] He, T., Cheng, J., Qin, J.-G., Li, Y. & Gao, T.-X. Comparative analysis of otolith morphology in three species of *Scomber*. *Ichthyol. Res.* **65**, 192–201. 10.1007/s10228-017-0605-4 (2018).

[93] Pothin, K., Gonzalez-Salas, C., Chabanet, P. & Lecomte-Finiger, R. Distinction between *Mulloidichthys flavolineatus* juveniles from Reunion Island and Mauritius Island (south-west Indian Ocean) based on otolith morphometrics. *J. Fish Biol.* **69**, 38–53. 10.1111/j.1095-8649.2006.01047.x (2006).

[94] Agüera, A. & Brophy, D. Use of saggital otolith shape analysis to discriminate Northeast Atlantic and Western Mediterranean stocks of Atlantic saury, *Scomberesox saurus saurus* (Walbaum). *Fish. Res.* **110**, 465–471. 10.1016/j.fishres.2011.06.003 (2011).

[95] Başusta, N. & Dürrani, Ö. Sexual dimorphism in the otolith shape of shi drum, *Umbrina cirrosa* (L.), in the eastern Mediterranean Sea: Fish size–otolith size relationships. *J. Fish Biol.* **99**, 164–174. 10.1111/jfb.14708 (2021). 33624838 10.1111/jfb.14708

[96] Bostanci, D. et al. Using otolith shape and morphometry to identify four *Alburnus* species (*A. chalcoides, A. escherichii, A. mossulensis* and *A. tarichi*) in Turkish inland waters. *J. Appl. Ichthyol.* **31**, 1013–1022. 10.1111/jai.12860 (2015).

[97] Nolf, D. G., Fischer and Verlag. *Handbook of paleoichthyology* Vol. 10 (Gustav, Fischer and Verlag, 1985).

[98] Gauldie, R. W. Function, form and time-keeping properties of fish otoliths. *Comp. Biochem. Physiol. A Mol. Integr. Physiol.* **91**, 395–402. 10.1016/0300-9629(88)90436-7 (1988).

[99] Reichenbacher, B., Sienknecht, U., Küchenhoff, H. & Fenske, N. Combined otolith morphology and morphometry for assessing taxonomy and diversity in fossil and extant killifish (*Aphanius, Prolebias*). *J. Morphol.* **268**, 898–915. 10.1002/jmor.10561 (2007). 17674357 10.1002/jmor.10561

